# A programmable platform enabling targeted chromosome substitution and cross-species stability profiling

**DOI:** 10.1093/procel/pwag010

**Published:** 2026-03-09

**Authors:** Lei Shi, Xiali Yang, Mingdi Wu, Chengye Zhao, Jun Wu, Erwei Zuo

**Affiliations:** State Key Laboratory of Genome and Multi-omics Technologies, Shenzhen Branch, Guangdong Laboratory of Lingnan Modern Agriculture, Key Laboratory of Gene Editing Technologies (Hainan), Ministry of Agriculture and Rural Affairs, Agricultural Genomics Institute at Shenzhen, Chinese Academy of Agricultural Sciences, Shenzhen 518120, China; State Key Laboratory of Genome and Multi-omics Technologies, Shenzhen Branch, Guangdong Laboratory of Lingnan Modern Agriculture, Key Laboratory of Gene Editing Technologies (Hainan), Ministry of Agriculture and Rural Affairs, Agricultural Genomics Institute at Shenzhen, Chinese Academy of Agricultural Sciences, Shenzhen 518120, China; State Key Laboratory of Genome and Multi-omics Technologies, Shenzhen Branch, Guangdong Laboratory of Lingnan Modern Agriculture, Key Laboratory of Gene Editing Technologies (Hainan), Ministry of Agriculture and Rural Affairs, Agricultural Genomics Institute at Shenzhen, Chinese Academy of Agricultural Sciences, Shenzhen 518120, China; State Key Laboratory of Genome and Multi-omics Technologies, Shenzhen Branch, Guangdong Laboratory of Lingnan Modern Agriculture, Key Laboratory of Gene Editing Technologies (Hainan), Ministry of Agriculture and Rural Affairs, Agricultural Genomics Institute at Shenzhen, Chinese Academy of Agricultural Sciences, Shenzhen 518120, China; Department of Molecular Biology, University of Texas Southwestern Medical Center, Dallas, TX 75235, United States; Hamon Center for Regenerative Science and Medicine, University of Texas Southwestern Medical Center, Dallas, TX 75235, United States; Cecil H. and Ida Green Center for Reproductive Biology Sciences, University of Texas Southwestern Medical Center, Dallas, TX 75235, United States; State Key Laboratory of Genome and Multi-omics Technologies, Shenzhen Branch, Guangdong Laboratory of Lingnan Modern Agriculture, Key Laboratory of Gene Editing Technologies (Hainan), Ministry of Agriculture and Rural Affairs, Agricultural Genomics Institute at Shenzhen, Chinese Academy of Agricultural Sciences, Shenzhen 518120, China

**Keywords:** CRISPR/Cas9, chromosome elimination, MMCT, chromosome substitution strains

## Abstract

Chromosome substitution strains (CSS) are critical tools for dissecting complex traits, although iterative breeding steps and intraspecific compatibility requirements limit conventional approaches. Here, we developed a Targeted chromosome Elimination And Microcell-mediated chromosome transfer platform (TEAM) for chromosome replacement combining CRISPR/Cas9-mediated chromosome elimination with microcell-mediated chromosome transfer (MMCT). Using this approach, we substituted the endogenous mouse Y chromosome (chrY) with either the mouse or human Y chromosome. Intraspecies substitutions yielded karyotypically stable embryonic stem cells that supported development into adult males. By contrast, in interspecies CSS, human chrY displayed severe instability and progressive DNA damage. Despite partial transcription of human chrY genes, recipient animals exhibited systemic inflammation, high rates of neonatal death, and poor growth. Reduced CENP-A levels were observed at human chrY centromeres, leading to segregation errors, micronuclei formation, and widespread chromosome rearrangements. This technology enables programmable construction of chromosome substitution models for investigating chromosomal function, genome evolution, and synthetic karyotype design in mammals.

## Introduction

Chromosome substitution strains (CSS), in which individual donor chromosomes are introduced into a defined genetic background, have emerged as powerful tools for dissecting complex biological traits (Nadeau et al., 2000). Originally designed for mapping quantitative trait locus (QTL), CSS have since been widely utilized in diverse research areas, including chromosome-specific gene regulation, epistatic interactions, disease modeling, and agricultural trait improvement in both animal and plant systems (Bellaloui et al., 2021; Kazuki and Oshimura, 2011; Miller et al., 2020; Nadeau et al., 2000; Shao et al., 2008; Singer et al., 2004; Wijnen et al., 2024; Xu et al., 2010).

Despite their broad utility, conventional CSS construction is constrained by technical and biological limitations, as the process depends on repeated backcrossing and marker-assisted selection over multiple generations, which is labor-intensive, time-consuming, and prone to unintended recombination (Flint et al., 2005; Nadeau et al., 2000). Moreover, traditional CSS strategies remain limited to intraspecific systems and cannot accommodate chromosomes from different species or synthetic origins for which reproductive or centromeric compatibility cannot be assumed (Henikoff et al., 2001; Kalitsis and Choo, 2012; Logsdon and Eichler, 2024). With rapid advances in synthetic genomics, humanized disease models, and interspecies reproductive engineering, the need for flexible, scalable, and breeding-independent strategies becomes increasingly urgent to keep pace with potential applications for CSS systems.

At present, new methods in genome editing and chromosome engineering have opened new avenues for developing more efficient and flexible chromosome substitution strategies. In particular, CRISPR/Cas9-mediated chromosome elimination has emerged as an effective tool for selectively removing entire chromosomes by targeting lineage-specific repetitive elements or centromeric sequences (Zuo et al., 2017). This strategy has been widely applied in a variety of research fields, such as elimination of extra chromosome 21 in cells from individuals with Down syndrome (Hashizume et al., 2025), assessment of chromosome loss in CRISPR/Cas9-engineered T cells (Tsuchida et al., 2023), and establishment of mosaic loss of Y chromosome (mLOY) disease models (Sano et al., 2022). Alternatively, microcell-mediated chromosome transfer (MMCT) offers a robust approach for delivering intact, natural, or engineered donor chromosomes into recipient cells via microcell fusion, and has been used to introduce native or artificial chromosomes in mammalian systems (Kazuki and Oshimura, 2011; Suzuki et al., 2020). Together, these technologies lay the foundation for overcoming temporal and biological constraints associated with conventional CSS production, enabling rapid, precise chromosome substitutions, even between different species.

Combining CRISPR/Cas9-mediated chromosome elimination with MMCT, we developed an optimized chromosome substitution platform that allows replacement of mouse Y chromosome (chrY) with a donor chrY of either mouse or human origin. We then applied this platform for *in vitro* and *in vivo* comparisons of intraspecies and interspecies chrY substitution strains to examine cross-species chromosome stability and the phenotypic consequences of chrY substitution in mice. We also investigated the potential mechanism underlying interspecies chrY instability. Beyond demonstrating the scalability and robustness of the platform for CSS construction across species, our study also highlights its utility as a functional assay for chromosomal incompatibility and centromere evolution, thus broadening the potential for CSS application in mammalian systems.

## Results

### Intraspecies Y chromosome substitution via TEAM

**Figure 1.**
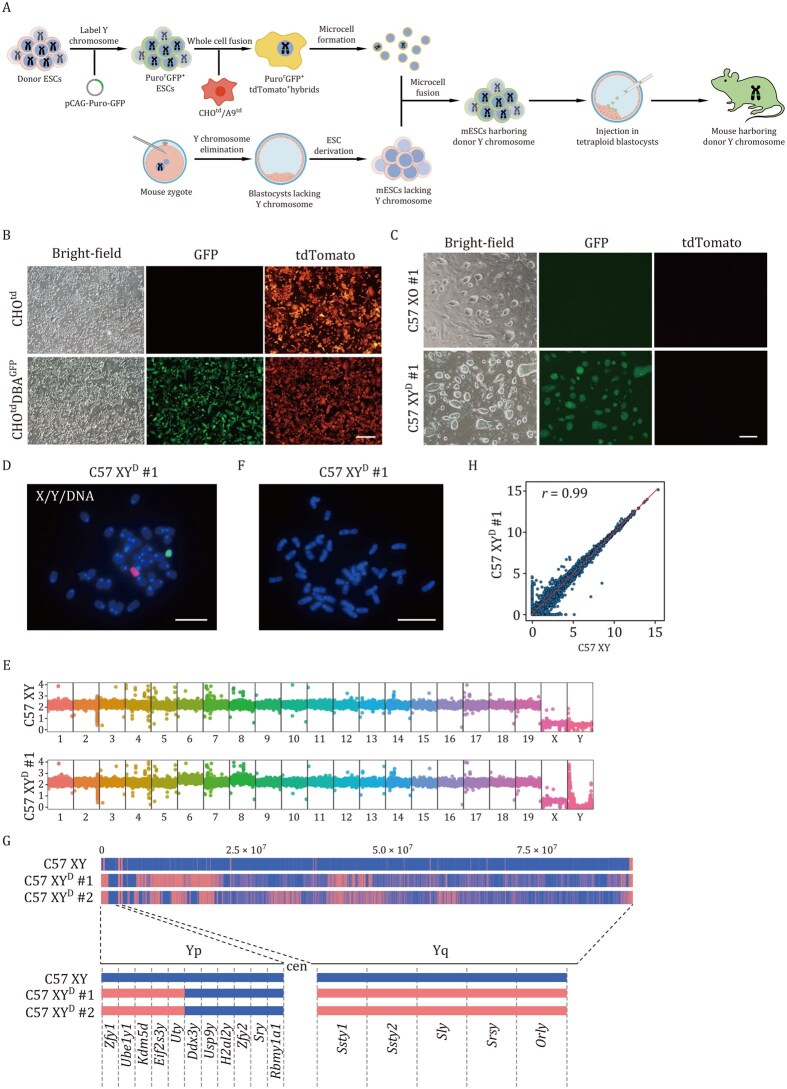
**Derivation of euploid C57 XY^D^ mESCs through mouse Y chromosome replacement**. (A) Stepwise strategy to introduce exogenous chromosomes without inducing aneuploidy. (B) Morphology of CHO^td^ cell line and CHO^td^DBA^GFP^ cell line. CHO^td^DBA^GFP^ cells harbor both the DBA-puro-GFP-originated GFP marker and the CHO^td^-originated tdTomato marker. Scale bar, 500 μm. (C) Morphology of C57 XO #1 and C57 XY^D^ #1 ESCs. C57 XY^D^ #1 cells exclusively harbor the DBA-puro-GFP-originated GFP marker, lacking the CHO^td^-originated tdTomato marker. Scale bar, 500 μm. (D) Representative DNA-FISH analysis of C57 XY^D^ #1 ESCs. Green, whole-chromosome probe for mouse Y chromosome; red, whole-chromosome probe for mouse X chromosome; blue, Hoechst-labeled DNA. Scale bar, 10 μm. (E) WGS analysis confirmed the presence of chrY in the C57 XY^D^ #1 ESC line. C57 XY, male C57BL/6 ESC line; vertical axis, copy number; horizontal axis, chromosome number. (F) Karyotyping of C57 XY^D^ #1 ESCs showed 40 chromosomes. Scale bar, 10 μm. (G) Mouse chrY content in male C57BL/6 (C57 XY), C57 XY^D^ #1 and C57 XY^D^ #2 ESC line. Top, WGS coverage profiles of the mouse chrY across three cell lines (C57 XY, C57 XY^D^ #1, and C57 XY^D^ #2) are displayed. The genomic positions (0.0–7.5 × 10^7^ bp) along the chrY are indicated at the top. Blue regions represent sequencing read coverage, confirming the presence of Y chromosomal DNA, while red regions denote coverage gaps. Bottom, cell line names are shown on the left and vertical bars that indicate the presence of the mouse chrY markers listed at the bottom. Markers are shown in order but are not spaced relative to their distance apart on chrY. Blue denotes the presence of the marker, red denotes a negative score, that is, the marker was not present. Yp, the short arm of chrY; Yq, the long arm of chrY; cen, centromere. (H) Comparison of gene expression values between C57 XY^D^ #1 ESCs and C57 XY ESCs. C57 XY, male C57BL/6 ESC line.

To overcome limitations of conventional CSS generation approaches, we developed the Targeted chromosome Elimination And Microcell-mediated chromosome transfer (TEAM) platform (Fig. 1A). As previous studies have shown that mouse artificial chromosomes (MACs) constructed from native chromosomes exhibit high stability in adult tissues and hematopoietic cells in mice (Kazuki et al., 2013; Takiguchi et al., 2014), we first examined the stability of CSS mice generated by TEAM using the mouse chrY as a proof of concept. We tagged the donor chrY by cotransfecting male DBA/2 embryonic stem cells (ESCs) with plasmids encoding Cas9, a donor construct containing a CAG-puromycin-GFP (CAG-puro-GFP) cassette flanked by homology arms, and an sgRNA targeting the region between *Uty* and *Ddx3y* (Fig. S1A–C). After selecting cells with puromycin, genomic PCR showed that 7 of the 18 clones (38.9%) were successfully targeted (Fig. S1D). Droplet digital PCR (ddPCR) confirmed single-copy integration of the selection marker at the expected locus in clones 9, 11, 15, and 17, without off-target recombination events (Fig. S1E). These clones, named DBA-puro-GFP, also showed strong GFP expression (Fig. S1F).

To obtain low-passage chrY recipient cell lines, Cas9 mRNA and two sgRNAs targeting spermiogenesis-specific transcript on Y 2 (*Ssty2*), which spans over 30 repeat sequences on the long arm of chrY, were injected into C57BL/6 zygotes (Fig. S2A). The treated embryos were then cultured *in vitro* to generate mouse ESCs (mESCs). Among the 11 resulting mESC clones, three lacked mouse chrY (Fig. S2B and S2C). These chrY-deficient clones, designated as C57 XO, exhibited a 39-chromosome karyotype with a single X chromosome (Fig. S2D and S2E), while the wild-type C57BL/6 mESCs harbor 40 chromosomes. Next, we fused DBA-puro-GFP #11 ESCs with CHO cells stably expressing tdTomato (CHO^td^) (Fig. 1B) to create hybrids (CHO^td^DBA^GFP^). Next, we utilized the conventional MMCT protocol to generate microcells. The CHO^td^DBA^GFP^ cells were treated with colchicine to induce micronucleation. Then, cells were treated with Latrunculin B to disrupt cytoskeletal integrity. Microcells were isolated by Percoll gradient centrifugation. The CHO^td^DBA^GFP^-derived microcells were then fused with C57 XO mESCs. Following puromycin selection, GFP-positive mESC clones (designated as C57 XY^D^) were obtained at a frequency of 1 × 10^−6^ (Fig. 1C). Fluorescence *in situ* hybridization (FISH)/whole genome sequencing (WGS) analysis indicated that a significant portion of chrY sequence could be detected in C57 XY^D^ mESCs (Fig. 1D and 1E). Notably, all C57 XY^D^ mESC lines exhibited a normal 40-chromosome karyotype (Fig. 1E and 1F). However, consistent with previous studies (O’Doherty et al., 2005), WGS analysis revealed substantial deletions on the transferred chrY. Specifically, C57 XY^D^ #1 showed ∼40.3% of chrY, while C57 XY^D^ #2 had a ∼38.3% deletion (Fig. 1G), and other C57 XY^D^ lines showed similar levels of chrY sequence loss.

Bulk RNA-seq analysis showed that C57 XY^D^ mESCs had transcriptomic profiles nearly identical to those of male C57BL/6 mESCs, with a strong correlation (Pearson *r *= 0.99 for C57 XY vs. C57 XY^D^ #1) (Fig. 1H). Combined with immunostaining for pluripotency markers OCT4, SOX2, and SSEA1, these results suggest that intraspecific chrY transfer had minimal effect on gene expression in mESCs (Fig. S3). The overall results support that the combined use of CRISPR/Cas9-mediated chromosome elimination and MMCT is a viable approach for intraspecies chrY substitution.

### MMCT-induced micronuclei formation drives DNA damage

Micronuclei (Crasta et al., 2012), small, separate nuclear structures formed when chromosomes lag during cell division, are both a sign and a cause of genomic instability. These structures are more fragile than normal nuclei, and their DNA often replicates out of sync with the rest of the cell, leading to DNA damage and chromosomal fragmentation (Liu et al., 2018; Zhang et al., 2015). In MMCT, colchicine is used to promote micronucleation formation. To assess how this step affects chromosome integrity within the MMCT workflow, we first examined the nuclear envelope stability of colchicine-induced micronuclei. We created a CHO^CN^ cell line that stably expresses mCherry-tagged nuclear localization signals (mCherry-NLS). These CHO^CN^ cells were fused with DBA-puro-GFP #11 mESCs to form CHO^CN^DBA^GFP^ hybrid cells. In colchicine-treated CHO^CN^DBA^GFP^ cells, we observed loss of mCherry-NLS signal in micronuclei, indicating nuclear envelope instability. In contrast, HBSS-treated control cells maintained the mCherry signal in cell nuclei (Fig. S4A). Additionally, immunostaining for γH2AX, a marker of DNA damage, revealed damage in 26.7% of micronuclei in colchicine-treated cells (Fig. S4B and S4C). WGS analysis further confirmed the extent of the damage: colchicine-treated CHO^td^DBA^GFP^ cells had 8,596 single nucleotide variants (SNVs) and 7,982 insertions/deletions (indels) not present in the control group (Fig. S4D). To further evaluate whether colchicine-induced DNA damage is dose- or time-dependent, we quantified γH2AX signals in CHO^td^DBA^GFP^ cells treated with varying colchicine concentrations (0, 50, 75, and 200 ng/mL) and exposure times (12–72 h). Prolonged exposure significantly increased DNA damage, with multiple conditions showing higher γH2AX signals at 72 h compared with 12 h. In CHO^td^DBA^GFP^ cells, higher colchicine doses also induced significantly greater DNA damage (Fig. S4E and S4F).

These findings demonstrate that MMCT, through colchicine-induced micronuclei formation, disrupts the nuclear envelope and causes widespread DNA damage in a dose- and time-dependent manner. This mechanism likely contributed to the chrY fragmentation seen in C57 XY^D^ mESCs.

### Generation of intraspecies Y-chromosome-replaced mice via tetraploid complementation

**Figure 2.**
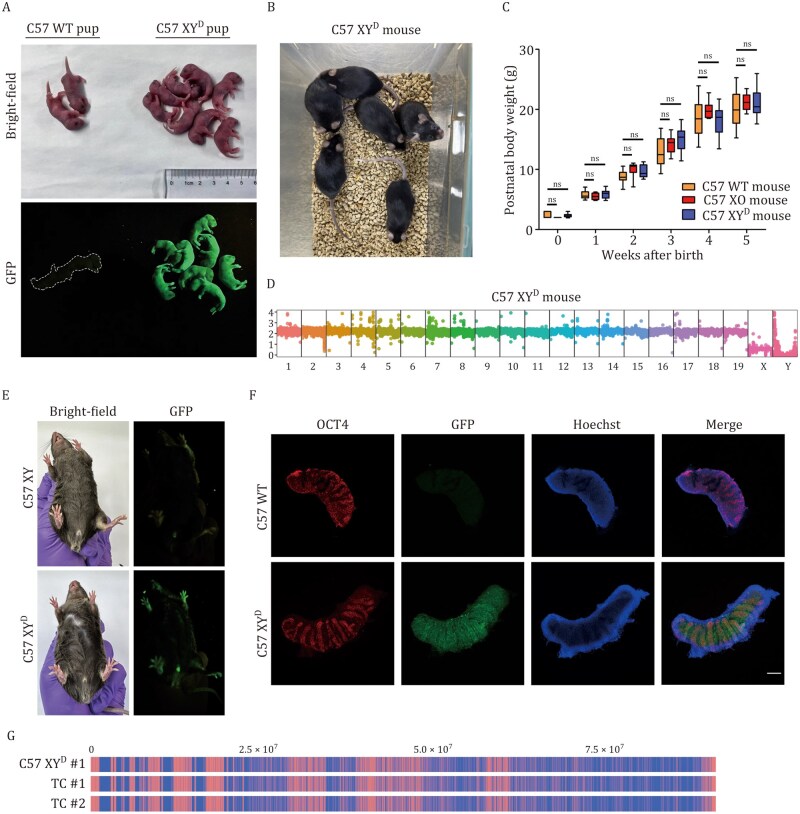
**Production of viable mice that carry intraspecies Y chromosome**. (A) Generation of full-term C57 XY^D^ pups. Photographs are shown in the top row, and images for GFP signals are shown in the bottom row. (B) Adult C57 XY^D^ mice. (C) Growth curves of wild-type C57BL/6 (*n* = 8), C57 XO (*n* = 6), and C57 XY^D^ (*n* = 8) mice. ns, no significance. (D) WGS analysis confirmed the presence of mouse chrY in the C57 XY^D^ mouse. Vertical axis, copy number; horizontal axis, chromosome number. (E) Adult C57 XY^D^ mouse showed male primary sex characteristics. The images for GFP signals are shown on the right. C57 XY mouse, male C57BL/6 mouse. (F) Immunofluorescence staining of E12.5 male C57BL/6 and C57 XY^D^ mouse genital ridges was shown, with OCT4 used as a marker for gonocytes. Scale bar, 200 μm. (G) WGS coverage profiles of the mouse chrY are displayed. The genomic positions (0.0–7.5 × 10^7^ bp) along the chrY are indicated at the top. Blue regions represent sequencing read coverage, confirming the presence of Y chromosomal DNA, while red regions denote coverage gaps. C57 XY^D^ #1, C57 XY^D^ #1 mESCs; TC #1, C57 XY^D^ mice #1; TC #2, C57 XY^D^ mice #2.

To test whether XY^D^ mESCs could be used to generate live animals, we injected C57 XY^D^ #1 mESCs into tetraploid B6D2F1 blastocysts. This approach successfully produced 28 viable, GFP-positive pups (Fig. 2A and Table S1), confirming the developmental competence of the modified mESCs. To evaluate the effects of chrY transfer, we compared the growth and development of C57 XY^D^ mice with wild-type C57BL/6 and C57 XO mice (generated using C57 XO mESCs). All groups showed normal growth into adulthood (Fig. 2B and 2C). WGS confirmed that the C57 XY^D^ mice carried the mouse chrY (Fig. 2D), and all developed as males (Fig. 2E). Histological analysis of E12.5 embryos revealed the presence of gonocytes in the genital ridges (Fig. 2F), indicating active and functional *Sry* gene expression (Wilhelm et al., 2007).

Further sequencing showed that the chrY DNA sequences in C57 XY^D^ mice closely matched those of the original mESCs (Fig. 2G), suggesting that the chromosome remained stable throughout development. The DNA deletions seen in the mESCs were therefore likely caused by the MMCT process itself, rather than developmental instability. These findings further reveal that although MMCT introduces micronucleus-associated DNA damage, such damage does not compromise the long-term stability of transferred chromosomes. Overall, these results demonstrate the feasibility of using the TEAM strategy for stable, intraspecific Y chromosome replacement in mice, and support its broader use for studying chromosome stability after chromosome transfer.

### TEAM enables replacement of mouse Y chromosome with human Y chromosome in mESCs

**Figure 3.**
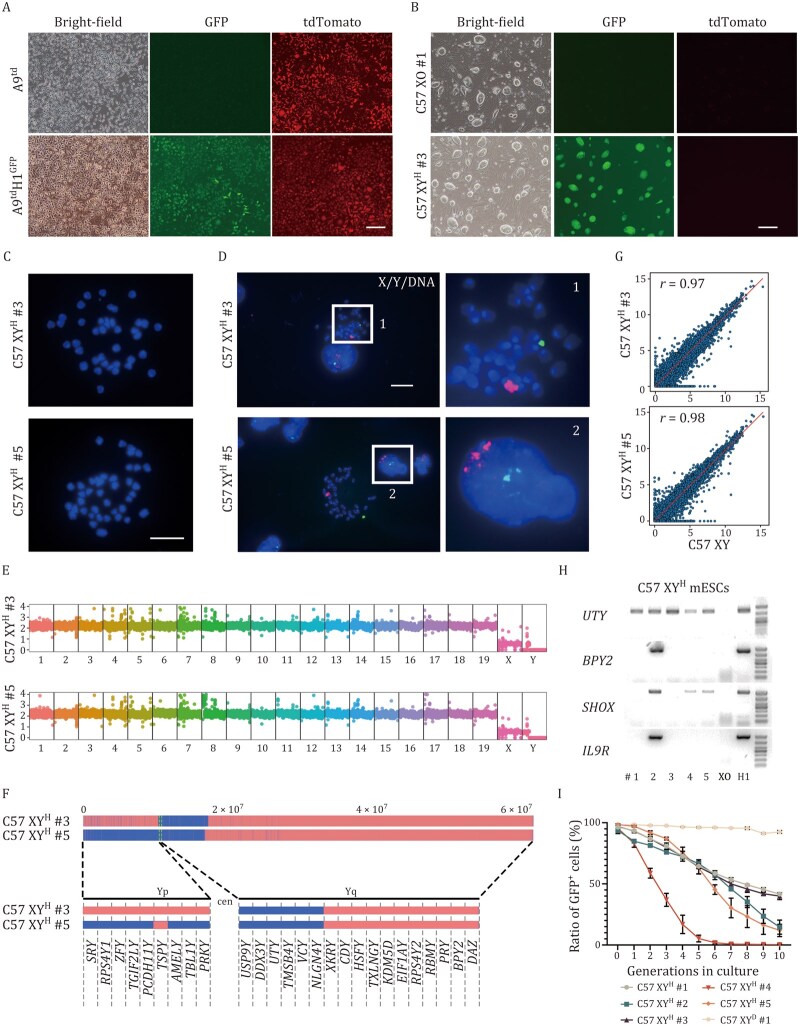
**Derivation of euploid C57 XY^H^ mESCs and evaluation of human chrY stability *in vitro***. (A) Morphology of A9^td^ cell line and A9^td^H1^GFP^ cell line. A9^td^H1^GFP^ cells harbor both the H1-puro-GFP-originated GFP marker and the A9^td^-originated tdTomato marker. Scale bar, 500 μm. (B) Morphology of C57 XO #1 and C57 XY^H^ #3 ESCs. C57 XY^H^ #3 cells exclusively harbor the H1-puro-GFP-originated GFP marker, lacking the A9^td^-originated tdTomato marker. Scale bar, 500 μm. (C) Karyotyping of C57 XY^H^ #3 and C57 XY^H^ #5 ESCs showed 40 chromosomes. Scale bar, 10 μm. (D) Representative DNA-FISH analysis of C57 XY^H^ #3 and C57 XY^H^ #5 ESCs. Green, whole-chromosome probe for human Y chromosome; red, whole-chromosome probe for mouse X chromosome; blue, Hoechst-labeled DNA. Scale bar, 10 μm. (E) WGS analysis confirmed the presence of human chrY in the C57 XY^H^ #3 and C57 XY^H^ #5 ESCs. Vertical axis, copy number; horizontal axis, chromosome number. (F) Human chrY content in C57 XY^H^ #3 and C57 XY^H^ #5 ESC line. Top, WGS coverage profiles of the chrY across two cell lines (C57 XY^H^ #3 and C57 XY^H^ #5) are displayed. The genomic positions (0.0–6 × 10^7^ bp) along human chrY are indicated at the top. Blue regions represent sequencing read coverage, confirming the presence of Y chromosomal DNA, while red regions denote coverage gaps. Bottom, cell line names are shown on the left and vertical bars that indicate the presence of the human chrY markers listed at the bottom. Markers are shown in order but are not spaced relative to their distance apart on chrY. Blue denotes the presence of the marker, red denotes a negative score, that is, the marker was not present. Yp, the short arm of chrY; Yq, the long arm of chrY; cen, centromere. (G) Comparative analysis of gene expression profiles between C57 XY^H^ #3 and C57 XY^H^ #5 ESCs relative to male C57 ESCs (C57 XY). (H) Genomic DNA PCR results of human chrY-specific genes (*UTY*, *BPY2*, *SHOX*, *IL9R*) in seven cell lines: C57 XY^H^ #1–5 (#1–5), C57 XO (XO), and human ESC H1 (H1). C57 XO served as a negative control (no human chrY), while H1 served as a positive control (intact human chrY). Variations of PCR results indicated distinct human chrY fragments retained in each C57 XY^H^ line. (I) Flow cytometry showed the ratio of GFP^+^ cells after serial culture without selection pressure in C57 XY^H^ #1–5 and C57 XY^D^ #1.

We next applied the TEAM platform to test cross-species chromosome replacement by substituting mouse chrY with human chrY. We first inserted a CAG-puro-GFP tag between two genes (*DDX3Y* and *UTY*) on the chrY in H1 human ESCs, creating three labeled clones (H1-puro-GFP; Fig. S5). Using one of these clones, we fused H1-puro-GFP #1 with tdTomato-expressing A9^td^ cells to create a donor cell line (A9^td^H1^GFP^) capable of providing microcells for chromosome transfer (Fig. 3A).

As with mouse chromosome transfers, colchicine-treated A9^td^H1^GFP^ cells showed signs of DNA damage, confirmed by γH2AX staining and WGS (Fig. S4). We then fused these donor microcells with C57 XO mESCs. After ­puromycin selection, 29 GFP^+^ mouse stem cell lines were recovered (Fig. 3B), at a frequency of 1–3 × 10^−6^. Karyotyping showed that 14 of these lines had a normal mouse chromosome number (40), while the rest had around 80 chromosomes, likely due to cell fusion events (Fig. 3C).

FISH analysis confirmed that the human chrY was successfully transferred and maintained as a separate chromosome in all 14 normal karyotype lines, now referred to as C57 XY^H^ mESCs (Fig. 3D). However, WGS revealed substantial deletions on the transferred human chrY: C57 XY^H^ #3 had lost ∼87.3% and C57 XY^H^ #5 had lost ∼72.6% of human chrY (Fig. 3E and 3F). Importantly, there were no copy number changes in mouse autosomes or the X chromosome, showing that only the transferred human chrY was affected. Despite these deletions, RNA-seq showed strong gene expression similarity between C57 XY^H^ mESCs and wild-type male C57 XY mESCs (Pearson *r *= 0.97 for XY^H^ #3 and 0.98 for XY^H^ #5; Fig. 3G). Immunostaining confirmed continued expression of key pluripotency markers (OCT4, SOX2, and SSEA1; Fig. S6). However, the human chrY showed poor stability over time. Flow cytometry revealed that in some C57 XY^H^ lines, GFP expression rapidly decreased with cell passages. By passage 7, nearly all C57 XY^H^ #4 cells had lost GFP. In contrast, C57 XY^H^ #3 and #5 retained 40% and 10% GFP^+^ cells after 10 passages, while mouse-derived C57 XY^D^ cells remained stable (Fig. 3H and 3I).

In summary, although replacing the mouse chrY with a human chrY had little effect on mESC self-renewal and gene expression, the human chrY was much less stable in mESCs after transfer.

### Neonatal death and poor growth in mice carrying the human Y chromosome

**Figure 4.**
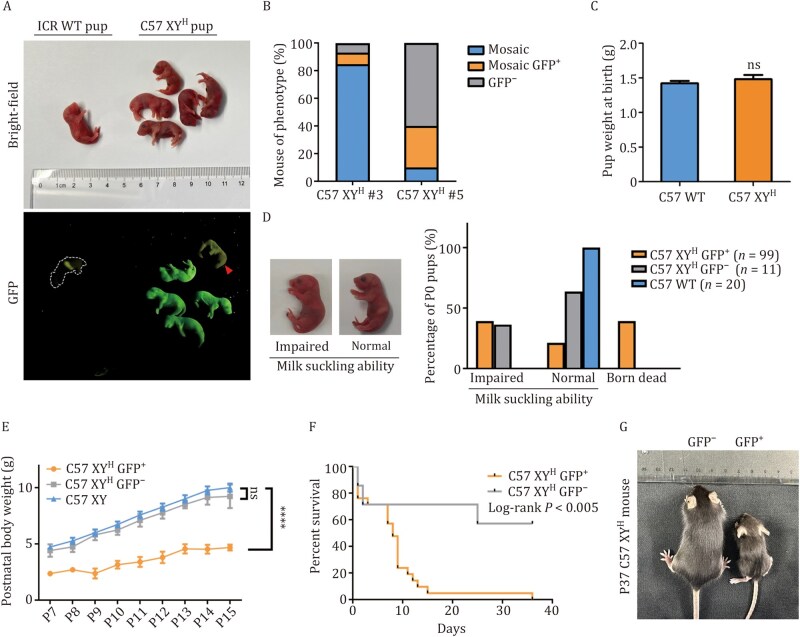
**Neonatal mortality and impaired growth in C57 XY^H^ mice**. (A) Generation of full-term C57 XY^H^ pups. Photographs are shown on the top, and images for GFP signals are shown on the bottom. The red arrow indicates a GFP^−^ C57 XY^H^ pup. (B) Composition of neonatal tetraploid complementation mice derived from C57 XY^H^ #3 and C57 XY^H^ #5 ESCs. Mice were categorized into three phenotypic groups based on GFP expression: GFP^+^ (fully GFP-positive, blue), mosaic GFP^+^ (partially GFP-positive, orange), and GFP^−^ (GFP-negative, gray). The proportions of each phenotype are shown for both ESC lines. (C) Body weights of wild-type C57BL/6 (*n* = 8) and C57 XY^H^ (*n* = 11) newborn pups. ns, no significance. (D) Left, 24 h after birth, neonatal pups with low amount or no milk in the stomach were classified as the impaired milk suckling group, while those with visible milk in the stomach were classified as the normal milk suckling group. Right, percentages of wild-type C57BL/6 (C57 WT; *n* = 20), C57 XY^H^ GFP^+^ (*n* = 99), and C57 XY^H^ GFP^−^ (*n* = 11) neonatal pups that were categorized into three phenotypic groups. (E) Postnatal body weight progression of three tetraploid complementation mouse groups: male C57BL/6 (C57 XY; *n* = 5), C57 XY^H^ GFP^+^ (*n* = 5), and C57 XY^H^ GFP^−^ (*n* = 5). Body weight was measured from P7 to P15. Data are presented as mean ± SEM. ****P < 0.0001, Repeated Measures ANOVA; ns, no significance. (F) Kaplan-Meier survival curves of C57 XY^H^ GFP^+^ (*n* = 21) and C57 XY^H^ GFP^−^ (*n* = 7) mice. The survival rate was monitored over 40 days. Statistical significance was determined using the log-rank test (*P* < 0.005). (G) Representative images of P37 C57 XY^H^ mice. The body size of GFP^+^ mouse was smaller compared with its GFP^−^ littermates (*n* = 4).

To test if mESCs with a human chrY (XY^H^ mESCs) could produce live animals, we injected C57 XY^H^ mESCs into tetraploid B6D2F1 blastocysts. This resulted in 122 pups (Fig. 4A; Table S1), showing that the human chrY did not prevent full-term embryonic development. However, unlike mice carrying the mouse chrY (C57 XY^D^), the C57 XY^H^ pups showed mixed GFP expression—some were GFP-positive, some GFP-negative, and others had patchy (mosaic) expression (Fig. 4A and 4B). Of the 112 pups from C57 XY^H^ #3, 87.5% were GFP^+^, 7.1% were GFP^−^, and 5.4% showed mosaic expression. In contrast, pups from C57 XY^H^ #5 were mostly GFP^−^ (60%). To rule out chimerism (the presence of mixed genetic background), we performed SNP genotyping and confirmed all pups carried only the C57BL/6 genome (Fig. S7), meaning the variation in GFP was due to instability or loss of the human chrY.

GFP^+^ C57 XY^H^ mice showed high neonatal death and poor growth after birth. At birth, body weights were similar across all groups (Fig. 4C). However, 39.3% of GFP^+^ pups had breathing issues or birth defects like omphalocele, and died soon after birth (Fig. 4D). Surviving pups raised by foster mothers showed poor suckling in both GFP^+^ and some GFP^−^ groups, leading to death from dehydration within 48 h. Only 21.2% of GFP^+^ and 63.6% of GFP^−^ pups had visible milk intake, compared with100% of wild-type mice (Fig. 4D; Table S1).

Pups that survived still showed growth problems. From postnatal day 7–15, GFP^+^ mice grew significantly slower than wild-type males, while GFP^−^ pups had slightly lower, but not significantly different, growth rates (Fig. 4E). Survival analysis showed GFP^−^ mice lived longer than GFP^+^ mice (Fig. 4F). Only one GFP^+^ mouse survived to day 37, weighing just 4.9 g—much smaller than its GFP^−^ littermates, which averaged 17.67 g (Fig. 4G).

These results show that replacing the mouse chrY with a human chrY causes serious health problems postnatally, including high death rates after birth and impaired growth.

### Transcriptomic alteration and inflammation in mice with a human Y chromosome

**Figure 5.**
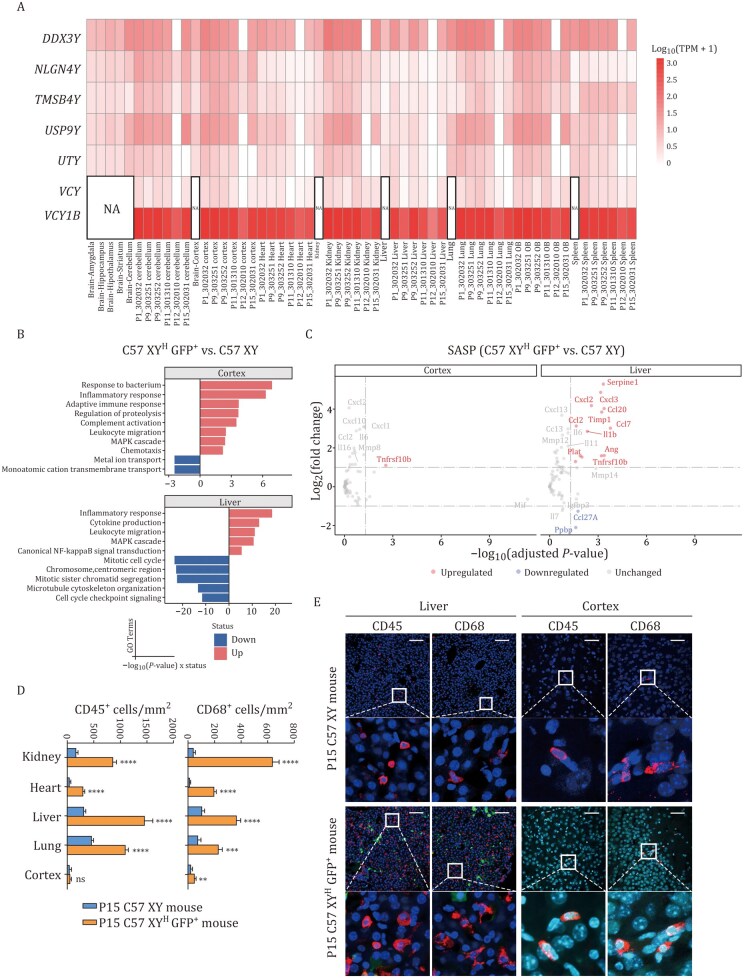
**Transcriptomic alteration and inflammation in C57 XY^H^ mice**. (A) The human chrY genes obtained from various tissues were selected for heatmap assay. The sample labeled by mouse age, identifier and organ (e.g., P9_303252 cerebellum) was extracted from C57 XY^H^ GFP^+^ mice aged P1–P15 (*n* = 6). The samples labeled solely by organ (e.g., Brain-Amygdala, Kidney) are derived from previously published data (Godfrey et al., 2020). OB, olfactory bulb. (B) Gene Ontology (GO) enrichment analysis of DEGs in the liver and cortex of C57 XY^H^ GFP^+^ mice versus male C57BL/6 mice (C57 XY). (C) Scatter plots showing the differential expression levels of SASP genes in the liver and cortex of C57 XY^H^ GFP^+^ mice compared with male C57BL/6 mice (C57 XY). (D) The density of CD45- and CD68-positive cells was quantified as the number of cells per mm^2^ (*n* = 20 each). Data are presented as mean ± SEM. **P < 0.01, ***P < 0.001, ****P < 0.0001, unpaired two-tailed Student’s *t* test. ns, no significance. C57 XY mouse, male C57BL/6 mouse. (E) Liver and cortex of P15 male C57BL/6 (C57 XY) and P15 C57 XY^H^ GFP^+^ mice were immunostained for CD45 or CD68 (red). Hoechst (blue) was used to label DNA. Scale bars, 50 μm.

Because the C57 XY^H^ GFP^+^ mice showed serious health issues, we examined how the human chrY affected their gene expression and immune response. We performed WGS and bulk RNA-seq on eight organs, including cortex, cerebellum, olfactory bulb, heart, liver, spleen, lung, and kidney, from six XY^H^ mice at different ages (P1 [*n *= 1], P9 [*n *= 2], P11 [*n *= 1], P12 [*n *= 1], and P15 [*n *= 1]). Variant calling in WGS data revealed deletions in three chrY genes, *DDX3Y*, *USP9Y*, and *UTY*, in all tissue samples of the P12 mouse, and *UTY* was deleted in all tissues of the P15 mouse. Other timepoints showed no major deletions in the seven key Y-linked genes we analyzed (*VCY1B*, *VCY*, *UTY*, *USP9Y*, *TMSB4Y*, *NLGN4Y*, and *DDX3Y*). As the deletions appeared across all tissues, they likely occurred early in development prior to organogenesis. The differences among XY^H^ mice suggest that these deletions were derived from random events. Subsequent RNA-seq analysis using the same samples confirmed that these human chrY genes were still expressed in all XY^H^ mice, although at varying levels. Expression of deleted genes like *DDX3Y* and *USP9Y* was lower, as expected. Interestingly, *VCY1B* was the most strongly expressed gene across tissues. When compared with human data (Godfrey et al., 2020), expression levels in mice were similar, suggesting the mouse cell environment supports normal expression of human chrY genes (Fig. 5A).

Next, we studied the overall changes in gene expression. Comparing XY^H^ and wild-type mice across tissues revealed 4,278 differentially expressed genes (DEGs), with the liver showing the most (1,335) and the cortex the fewest (121). Many of the upregulated genes were related to inflammation, immune cell movement, and cytokine ­production. Genes linked to the senescence-associated secretory phenotype (SASP)—such as *Il1a, Il1b, Il6, Ccl2,* and *Cxcl2*—were also significantly increased (Figs. 5B, 5C, S8B and S8C). In the liver, downregulated genes were mostly involved in cell division and cytoskeleton organization, suggesting impaired cell growth (Fig. 5B). Consistent with the transcriptomic data, immune cells, CD45^+^ lymphocytes and CD68^+^ macrophages, were more abundant in the liver, heart, kidney, and lungs of XY^H^ mice than in wild-type controls. Only the cortex showed a mild but noticeable increase in macrophages (Figs. 5D, 5E, and S8D).

In summary, our results demonstrate that the transferred human chrY experienced random gene deletions and triggered widespread changes in mouse transcriptome. These included strong inflammatory responses and immune cell infiltration across multiple organs, likely contributing to the health problems observed in C57 XY^H^ GFP^+^ mice.

### Instability of human Y chromosome in mice

**Figure 6.**
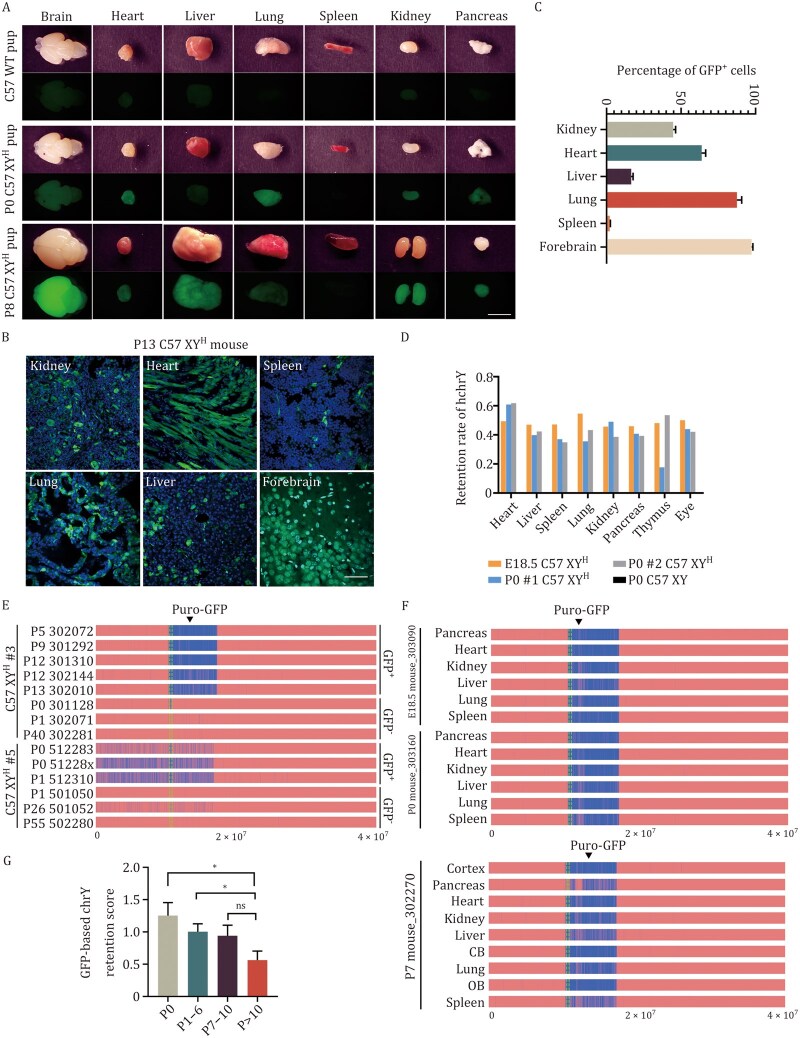
**Variable GFP expression and Y chromosomal instability in C57 XY^H^ mice**. (A) Morphology of different organs in wild-type C57BL/6 (C57 WT), P0 C57 XY^H^ and P8 C57 XY^H^ pups. The images for GFP signals are shown in the bottom row. Scale bar, 5 mm. (B) Representative images showing the percentage of GFP^+^ cells among different tissues in P13 C57 XY^H^ mouse. Scale bar, 400 μm. (C) Percentages of GFP^+^ cells among different tissues in P13-P15 C57 XY^H^ mice (*n* = 20 per group). (D) Retention of human chrY in different organs of C57 XY^H^ (E18.5 C57 XY^H^, P0 #1 C57 XY^H^, P0 #2 C57 XY^H^) and male C57BL/6 (P0 C57 XY), which was determined by real-time genomic DNA PCR of gene *UTY* on human chrY. (E) Human chrY content in tail genomic DNA of C57 XY^H^ #3 and C57 XY^H^ #5 mice. Mouse identifiers (IDs) are listed on the left. WGS coverage profiles of human chrY across 14 mice are shown, with genomic positions (0.0–6 × 10^7^ bp) annotated at bottom. Blue regions represent sequencing read coverage, confirming the presence of Y chromosomal DNA, while red regions denote coverage gaps. The Puro-GFP knock-in site on human chrY is labeled. Corresponding GFP phenotypes for individual mice are displayed on the right. (F) Human chrY content in genomic DNA from various tissues of C57 XY^H^ #3 mice. Mouse identifiers (IDs) and the age of each mouse (E18.5, P0 and P7) are listed on the left. WGS coverage profiles of human chrY across different tissues are shown, with genomic positions (0.0–6 × 10^7^ bp) annotated at bottom. Blue regions represent sequencing read coverage, confirming the presence of Y chromosomal DNA, while red regions denote coverage gaps. The Puro-GFP knock-in site on human chrY is labeled. All these mice are GFP^+^. CB, cerebellum; OB, olfactory bulb. (G) GFP-based chrY retention levels were quantified using WGS data from tail genomic DNA of C57 XY^H^ mice. For each animal, a GFP-based chrY retention score was calculated as the ratio between the average read depth across the GFP integration region and that of the autosomal reference gene *Gapdh* (Retention score = GFP depth/Gapdh depth). Mice were grouped according to postnatal survival time (P0, *n* = 5; P1–6, *n* = 8; P7–10 *n* = 4; P > 10, *n* = 5). Data are presented as mean ± SEM. *P < 0.05, unpaired two-tailed Student’s t test. ns, no significance.

Next, we examined how the human chrY behaves across different stages and tissues during mouse development. In C57 XY^H^ GFP^+^ pups, GFP (used to trace the human chrY) was detected in most major organs, including the brain, heart, liver, lungs, kidneys, and pancreas. The spleen, however, showed little to no signal (Fig. 6A). When analyzing tissue sections, we found significant variation in the proportion of GFP^+^ cells between organs, with the forebrain showing the highest levels (Figs. 5E, 6B, 6C, and S8D). Real-time PCR targeting the human *UTY* gene confirmed this uneven distribution and showed that expression levels also varied within individual organs (Fig. 6D).

WGS analysis of both GFP^+^ and GFP^−^ XY^H^ mice revealed that in GFP^−^ animals, the human chrY was either completely lost or present only in fragments (Fig. 6E). In contrast, GFP^+^ mice retained the human chrY, but it showed different degrees of DNA damage depending on the tissue and the individual (Fig. 6E and 6F). These results are consistent with earlier observations of inconsistent gene expression from the transferred chrY (Fig. 5A). Further comparisons at different developmental timepoints revealed that damage to the human Y chromosome increased with age and was not uniform across tissues (Fig. 6F and 6G).

Altogether, these results show that the human chrY, unlike mouse chrY, when introduced into mice via MMCT, undergoes persistent DNA damage and progressive instability *in vivo*.

### Structural instability and complex rearrangements in transferred human Y chromosome

**Figure 7.**
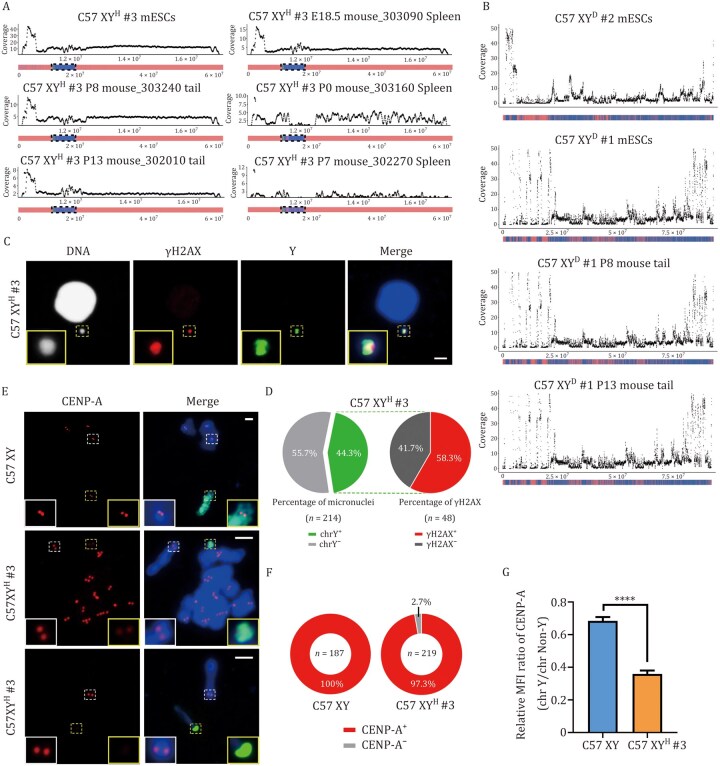
**Persistent rearrangements in the transferred human Y chromosome**. (A) Whole-genome sequencing (WGS) analysis of C57 XY^H^ #3 mESCs and C57 XY^H^ #3 mouse tissues carrying complex human chrY rearrangements exhibiting oscillating DNA copy-number patterns. Human Y-chromosome coverage was calculated using a 50 kb window with a 10 kb step size, while mouse Y-chromosome sequencing depth was computed using 100 bp bins. Each panel represents an individual sample (mESCs, P8 mouse_303240 tail, P13 mouse_302010 tail, E18.5 mouse_303090 spleen, P0 mouse_303160 spleen, and P7 mouse_302270 spleen). Black dots denote sequencing depth across the mappable regions of chrY (horizontal axis). The WGS coverage profile of chrY is shown below the axis: blue regions indicate sequencing read coverage confirming the presence of Y-chromosomal DNA, while red regions denote coverage gaps. The dashed black box highlights the chromosomal region corresponding to the scatter plot above. (B) WGS analysis of C57 XY^D^ mESCs and C57 XY^D^ mouse tails with complex mouse chrY rearrangements. Coverage was calculated using a 50 kb window with a 10 kb step size, while mouse Y-chromosome sequencing depth was computed using 100 bp bins. Each panel represents an individual sample (C57 XY^D^ #1 mESCs, C57 XY^D^ #2 mESCs, P8 C57 XY^D^ #1 mouse tail, and P13 C57 XY^D^ #1 mouse tail). (C) Representative Immuno-FISH images of micronucleated C57 XY^H^ #3 mESCs labeled for DNA (Hoechst, white), γH2AX (red) and human chrY (green). Insets show enlarged images of micronuclei. Scale bar, 5 μm. (D) Proportion of micronuclei containing human chrY and their γH2AX status in C57 XY^H^ #3 mESCs. The left pie chart shows the distribution of human chrY within micronuclei, revealing that 44.3% of the total detected micronuclei (*n* = 214) contained chrY (chrY^+^), while the remaining 55.7% lack chrY (chrY^−^). The right pie chart represents the percentage of γH2AX-positive (γH2AX^+^, 58.3%) and γH2AX-negative (γH2AX^−^, 41.7%) micronuclei among those carrying human chrY (*n* = 48). (E) Representative Immuno-FISH images of metaphase spreads prepared from male C57 and C57 XY^H^ #3 mESCs labeled for DNA (Hoechst, blue), CENP-A (red) and chrY (green). Insets provide magnified views of the regions delineated by the dashed boxes, each corresponding to their respective colors. Scale bars, 5 μm. (F) Proportion of CENP-A^+^ chromosome in male C57BL/6 (C57 XY) and C57 XY^H^ #3 mESCs. Numbers in the middle of the ring indicate the number of sights analyzed per experiment. (G) Mean fluorescence intensity of CENP-A on chrY is normalized against the autosome or chrX centromere. Data are presented as mean ± SEM. ****P < 0.0001, unpaired two-tailed Student’s *t* test (C57 XY, *n* = 30; C57 XY^H^ #3, *n* = 64).

To further investigate the instability of the transferred human chrY, we analyzed structural changes and copy number alterations using WGS data from C57 XY^H^ #3 mESCs (passage 9), tail samples from two C57 XY^H^ #3 mice (P8 and P13), and spleen tissues from three mice at different ages (E18.5, P0, and P7) (Figs. 3F, 6E, and 6F). Compared with mESCs, tail samples showed additional rearrangements in human chrY, and spleens exhibited even more complex and extensive copy number alterations (Fig. 7A). In contrast, no such changes were seen in mouse chrY from control C57 XY^D^ #1 mice (Fig. 7B), highlighting the specificity of these alterations to the human chromosome.

These patterns of instability, including copy number variations, are often linked to DNA damage from errors in chromosome segregation and micronuclei formation (Zhang et al., 2015). Supporting this, FISH analysis combined with γH2AX staining showed that 44.3% of micronuclei in C57 XY^H^ mESCs contained human chrY, and 58.3% of those were γH2AX-positive, indicating DNA damage (Fig. 7C and 7D). This aligns with RNA-seq findings of downregulated genes related to the mitotic cycle, microtubule structure, and checkpoint signaling in the liver (Fig. 5B and Table S4). Centromere function is crucial for correct chromosome segregation. Although human chrY includes repetitive alphoid DNA, it lacks functional CENP-B boxes (Earnshaw et al., 1987; Fachinetti et al., 2015; Ly et al., 2017). To assess centromere activity, we measured CENP-A (a key centromere protein) intensity at the human chrY in C57 XY^H^ mESCs and compared it with mouse chrY in C57BL/6 cells. After normalizing for total CENP-A levels per cell, we found that ∼2.7% (6/219) of human chrY lacked detectable CENP-A signal (Fig. 7E and 7F). Further FISH analysis revealed that ∼83.3% (5/6) of these chromosomes were involved in interchromosomal rearrangements, containing segments from both human chrY and mouse chromosomes (Fig. 7E). Overall, the human chrY showed significantly weaker CENP-A signal compared with native mouse chrY (Fig. 7E and 7G), consistent with earlier reports that low CENP-A leads to mis-segregation (Fachinetti et al., 2015; Ly et al., 2017).

These findings indicate that transferring human chrY into mice results in ongoing chromosomal instability, marked by structural rearrangements, persistent DNA damage, and variation between tissues. Defective centromere formation, due to reduced or absent CENP-A and inter-chromosomal fusions, likely causes segregation errors and micronuclei formation, further driving the instability of human chrY in a mouse background. These insights reinforce the importance of centromere compatibility in construction of karyotype-stable interspecies CSS and highlight TEAM platform’s utility for assessing chromosomal barriers across species.

## Discussion

This study establishes TEAM, a modular chromosome substitution platform that integrates CRISPR/Cas9-mediated chromosome elimination with MMCT to enable targeted replacement of endogenous chromosomes in mammalian cells. Using this platform, we demonstrate proof-of-concept replacement of the Y chromosome in both intra- and interspecies contexts, providing a foundation for systematic exploration of chromosome-scale engineering strategies.

Using this system, we successfully substituted endogenous chrY in C57BL/6 mESCs with an exogenous chrY from either DBA/2 or H1 ESCs. In intraspecies CSS, all mice generated via tetraploid complementation (C57 XY^D^) displayed uniform GFP expression, and WGS analysis confirmed that these animals harbored a chrY nearly identical to that in the original mESCs. Although partial DNA damage was detected on the transferred Y chromosome, transcriptomic analysis revealed no significant differences between C57 XY^D^ and male C57BL/6 mESCs. Two factors may contribute to this observation. First, the mouse chrY encodes a limited number of protein-coding genes, and the successful generation of tetraploid complementation mice from Y-null mESCs (Fig. 2C) indicates that these genes play only a minor role in maintaining pluripotency. Second, the transcriptomic similarity suggests that the TEAM-mediated chromosome substitution process does not cause extensive perturbations to the global gene expression landscape or to other chromosomes within the chrY recipient cells. The successful replacement of mouse chrY and generation of viable offspring underscore the TEAM system’s developmental compatibility. In contrast, our tests involving interspecies chrY replacement resulted in pronounced genomic instability, developmental failure, and postnatal lethality, despite detectable expression of human Y-linked genes. These different outcomes between intra- and interspecies CSS highlight the power of TEAM to enable efficient chromosome substitution while also allowing dissection of the boundaries of chromosomal compatibility among species.

Mechanistically, the instability of human chrY in the murine background is driven, in part, by reduced levels of the histone H3 variant, CENP-A, at the human centromere, a key determinant of centromere identity (McKinley and Cheeseman, 2016). This phenomenon aligns well with prior evidence showing that insufficient CENP-A disrupts kinetochore formation and leads to chromosome mis-segregation (Fachinetti et al., 2015; Ly et al., 2017). Both centromere DNA sequence and protein components, including CENP-A, are rapidly evolving (Maheshwari et al., 2015; Malik and Henikoff, 2001). In humans, centromeres are composed mainly of 171-base pair α-satellite higher-order repeats, whereas centromeres in mice consist of more uniform 120-bp minor satellite repeats, surrounded by less-ordered 234-bp major satellite arrays (Henikoff et al., 2001; McKinley and Cheeseman, 2016; Rattner, 1991). Apart from differences in DNA sequence, human CENP-A shares less than 60% sequence identity with canonical H3 and shows significant divergence across species, especially within its highly variable N-terminal domain (Stirpe and Heun, 2023). Molecular conflicts disrupting centromere maintenance have been reported to induce Xenopus hybrid inviability (Kitaoka et al., 2022). These data suggest that evolutionary divergence between species may impair murine CENP-A binding on human chrY in mouse cells, contributing to its instability after transfer.

We observed severe biological consequences of chrY instability at both the cellular and organismal levels. In ESCs and mouse tissues, human chrY was progressively lost or rearranged, while animals carrying the substituted chrY exhibited systemic inflammation, postnatal growth retardation, and perinatal lethality. As DNA damage and genomic instability are known to activate immune and senescence pathways, for example, cGAS-STING, NF-κB, and ATM signaling (Pezone et al., 2023; Wu et al., 2024; Zhao et al., 2023), we propose that the instability of the transferred chrY triggers chronic inflammation, which subsequently drives the observed developmental defects. Interestingly, although we found that several genes on human chrY were highly expressed in the mouse cortex, no significant increase in inflammatory markers was detected in this brain region of C57 XY^H^ GFP^+^ males compared with wild-type males. Notably, activated T cells are known to cross the blood–brain barrier under a variety of inflammatory or antigen-driven conditions, including autoimmune and neurodegenerative states (Chen et al., 2023; Goverman, 2011). Thus, the absence of immune infiltration in the cortex, despite robust chrY gene expression and the highest retention of the transferred chrY among all examined tissues, indicates that chrY-derived proteins alone are insufficient to elicit an adaptive immune response. Rather, it is likely the genomic instability and resulting cellular stress from the damaged human chrY drive the pathological response. These findings highlight the importance of maintaining chromosomal integrity in interspecies CSS and further reinforce the link between chromosome instability and inflammatory disease phenotypes. In addition, many phenotypes of C57 XY^H^ mouse are also characteristic of mice with imprinting abnormalities (Li et al., 2025). This raises the possibility that defects in genomic imprinting within the C57 XY^H^ mESCs may contribute, at least in part, to the observed developmental and postnatal abnormalities. Future studies should systematically evaluate DNA methylation and allelic expression at key imprinting regions in C57 XY^H^ mESCs to clarify the relationship between human chrY substitution and potential genomic imprinting disorders.

Interestingly, earlier studies involving mice carrying native human chromosomes or human artificial chromosomes (HACs) did not report the same level of developmental defects or postnatal mortality that we observed in the current study (Suzuki et al., 2006; Takiguchi et al., 2014). This difference likely reflects fundamental variations in the stability of transferred chromosomal material. Previous work has mainly focused on autosomes or autosome-derived HACs (Carroll et al., 2010), which contain both CENP-A and CENP-B centromeric binding sites (Carroll et al., 2010; Fachinetti et al., 2015). CENP-B binds to specific 17-base-pair repetitive DNA motifs known as CENP-B boxes. This interaction enhances the fidelity and stability of human centromere function (Fachinetti et al., 2015). In contrast, human chrY lacks CENP-B boxes, making it inherently more prone to segregation errors and DNA damage (Fachinetti et al., 2015). As a result, despite some degree of instability reported in transferred human autosomes or HACs in mouse models, the genomic stress they cause is typically not severe enough to trigger widespread inflammation or developmental failure such as we observed with human chrY transfer. Our findings emphasize that the structural features of a transferred chromosome, especially at the centromere, are critical determinants of its long-term stability and biological impact.

Notably, although chrY was used for proof-of-concept demonstration, future application of TEAM to autosomes, X chromosome, artificial chromosomes, or synthetic constructs will require overcoming additional chromosome-specific barriers. These include centromere compatibility, gene dosage balance, and preservation of pluripotency following large-scale genomic perturbation. Systematic exploration of these constraints will be necessary to establish the broader utility of chromosome substitution technologies.

Several limitations of our current study warrant consideration. First, MMCT involves colchicine treat­ment and micronuclei formation, which can cause genome-wide DNA damage, as we observed via γH2AX staining and structural variant analysis. Therefore, protocol optimization or exploring micronucleation-independent chromosome transfer approaches may partially mitigate procedure-induced DNA damage. Isolated metaphase chromosome transfer (iMCT) and flow-sorted chromosome transfer (FSCT) have been reported to function without relying on micronucleation (de Jong et al., 2001; Klobutcher et al., 1980; Suzuki et al., 2010). Furthermore, an improved version of MMCT (R-MMCT) has been reported (Petris et al., 2025). In this method, colchicine exposure time was shortened from 48–72 h to 7–9 h. This modification prevents micronucleus formation during chromosome preparation and markedly reduces DNA damage. These findings provide an important conceptual framework for future refinement of our TEAM platform. Improving the integrity of the nuclear envelope and optimizing centromere compatibility, potentially achieved via artificial centromere engineering or chimeric CENP-A rescue, may enhance the fidelity of interspecies chromosome transfer. Additionally, future studies will further explore the adaptability of the TEAM platform to other autosomes or X chromosome.

In conclusion, the TEAM platform establishes a framework for constructing CSS models. While we acknowledge that the current procedure may introduce DNA damage to the recipient chromosome, the system provides a critical proof of concept for targeted replacement of chromosomes, or at least chromosomal fragments, in mammalian cells. Notably, our work represents the first successful interspecies chromosome substitution, demonstrating the feasibility of cross-boundary chromosomal engineering. Despite the technical challenges, TEAM offers a versatile and scalable toolkit that opens new avenues for investigation of chromosome biology across species.

## Supplementary Material

pwag010_Supplementary_Data

## Data Availability

The raw sequence data are available from the National Center for Biotechnology Information Sequence Read Archive database under the accession code PRJNA1259728. All raw sequence data are also available from the China National GenBank DataBase under accession number CNP0007331.

## Code availability

No specific custom script was used in this study, and if applicable, will be accessed with reasonable requests.
