# Supplementary material for: A programmable platform enabling targeted chromosome substitution and cross-species stability profiling
Source: Protein Cell. 2026 Mar 9;17(6):528–42. doi: 10.1093/procel/pwag010 (PMC13225725; doi:10.1093/procel/pwag010)
Supplement: pwag010_Supplementary_Data [file pwag010_supplementary_data.zip › PAC-25855-ZEW-Supplementary Materials.docx]

**Supplemental Materials**

**Methods**

**Animals**

C57BL/6 female mice aged 4 weeks and ICR (CD1) female mice aged 8 weeks were purchased from Guangdong Medical Laboratory Animal Center. 8-week-old B6D2F1 female mice and 8-week-old DBA/2 male mice were purchased from Vital River Laboratory (Beijing). Mice were housed in a 12-h light/dark cycle at 20 – 24 °C and 40 – 60% humidity. All animals involved in the study were cared for in accordance with the guidelines established by the Biomedical Research Ethics Committee of Agricultural Genomics Institute in Shenzhen, Chinese Academy of Agricultural Sciences.

**Cell culture**

The mouse embryonic fibroblasts, A9, A9^td^, A9^CN^, A9^CN^H1^GFP^ and A9^td^H1^GFP^ cell lines were grown in Dulbecco’s modified Eagle’s medium (DMEM; YEASEN) supplemented with 10% fetal bovine serum (FBS; Biological Industries).

The CHO, CHO^td^, CHO^CN^, CHO^CN^DBA^GFP^ and CHO^td^DBA^GFP^ cell lines were maintained in Ham’s F-12 nutrient mixture (Gibco) containing 10% FBS (Gibco).

C57BL/6 and DBA/2 mESCs were cultured on irradiated mouse embryonic fibroblasts. Cells were passaged every 3 - 4 days with Trypsin (Gibco) at a split ratio of 1:8 - 1:10. The components of the mouse ESC culture medium (2iL) are: Dulbecco's modified Eagle medium (DMEM) (Millipore), 15% FBS (Gibco), 1 × minimal essential medium (MEM) nonessential amino acids solution (Gibco), 1 × GlutaMAX (Gibco), 1 × Nucleosides (Gibco), 1 × β-mercaptoethanol (Gibco), 1 μM PD0325901 (Selleck), 3 μM CHIR99021 (Selleck), and 1000 units/mL mouse LIF (Millipore).

Human ES cell line H1 (WA01, Passages 25-45) were kindly provided by Prof Xiaoqing Zhang (Tongji University, Shanghai, China). H1 ESCs were cultured on Matrigel (Corning)-coated dishes in ncTarget (nuwacell) and passaged every 5 – 7 days through dispase (Gibco) digestion.

Mycoplasma contamination test was performed weekly.

***In vitro* transcription of Cas9 mRNA and sgRNAs**

To prepare Cas9 mRNA, the T7 promoter sequence was added to the Cas9 coding region by PCR amplification of px260 (Addgene, 42229) using the primer pair listed in Table S2A. The T7-Cas9 PCR product was purified using Gel Extraction Kit (Omega) and used as the template for *in vitro* transcription (IVT) of Cas9 mRNA using the mMESSAGE mMACHINE T7 kit (Life Technologies).

To prepare sgRNAs, the T7 promoter sequence was added to the sgRNA template by PCR amplification of px330 (Addgene, 42230) using the primer pair listed in Table S2A. The T7-sgRNA PCR product was purified using Gel Extraction Kit (Omega) and used as the template for IVT of sgRNAs using the MEGAshortscript T7 kit (Life Technologies). Both the Cas9 mRNA and sgRNAs were purified using the MEGAclear kit (Life Technologies) and eluted with elution buffer according to the standard protocol.

**Microinjection of mRNAs into zygotes**

Super ovulated B6D2F1 female mice (8 weeks old) were mated with B6D2F1 male mice (2 – 6 months old). Zygotes were collected at E0.5 (the presence of a virginal plug was defined as E0.5) in M2 medium (Millipore) and cultured in KSOM+AA with D-Glucose (Millipore). The mixture of Cas9 mRNA (100 ng/μl) and sgRNAs (100 ng/μl) was injected into the cytoplasm of zygotes with well recognized two pronuclei in a droplet of M2 medium containing 5 μg/ml cytochalasin B (CB, Sigma-Aldrich) using a FemtoJet microinjector (Eppendorf) with continuous flow settings.

**Derivation of mESCs**

Mouse blastocysts from C57BL/6 and DBA/2 were used for mESCs derivation. In brief, zona pellucida was removed with Tyrode’s solution (Sigma-Aldrich), and each blastocyst was transferred into a 96-well plate and cultured on mouse embryonic fibroblasts (MEFs) with 2iL medium. After 5 – 7 days in culture, the outgrowths of blastocysts were disaggregated and replated onto newly prepared MEFs.

**Plasmid construction and genome editing**

mCAG-Puro-GFP plasmid for generation of DBA-puro-GFP cell line was comprised of homology arms span intergenic region between *Uty* and *Ddx3y* (chrY: 1259028-1260324 GRCm39/mm39 mouse assembly) and a CAG-puro-GFP cassette. mCAG-Puro-GFP plasmid, plasmids encoding Cas9 and sgRNA1 were transfected into dissociated DBA/2 mouse ESCs using Lipofectamine 3000 (Thermo Fisher Scientific) following the protocol recommended by the manufacturer.

hCAG-Puro-GFP plasmid for generation of H1-puro-GFP cell line contains homology arms span intergenic region between *DDX3Y* and *UTY* (chrY: 13095712-13096644 GRCh38/hg38 human assembly) and a CAG-puro-GFP cassette. hCAG-Puro-GFP plasmid was electroporated into human ES cell line H1 (WA01) with Cas9 plasmid and sgRNA4 as described(Chi et al., 2017).

48 h post-transfection, both DBA/2 and H1 ESCs were treated with 1 μg/mL puromycin (InvivoGen). Drug-resistant colonies were manually picked between 5 and 7 days and further expanded clonally.

To generate CHO^td^ and A9^td^ cell lines, PBL-CAG-tdTomato-PBR and CMV-PBase were introduced into CHO and A9 cells with Lipofectamine 3000 (Thermo Fisher Scientific) using the manufacturer’s instructions, according to a previous report(Huang et al., 2024). 5 – 7 days after transfection, cells were sorted out by BD FACS Aria II and cultured into independent cell lines.

The mCherry-NLS plasmid containing three copies of the SV40 LT NLS as reported(Micutkova et al., 2012). To generate CHO^CN^ cell line, CHO cells were transfected with pmCherry–NLS using Lipofectamine 3000 (Thermo Fisher Scientific) and isolated through cell sorting.

**Genotyping**

Genomic DNA of mouse blastocysts and ESCs were extracted using lysis buffer containing proteinase K (Vazyme). Genomic DNA of mouse tissues were extracted using DNeasy Blood & Tissue Kit (QIAGEN) and diluted using ddH_2_O. Genomic PCRs were performed using 2 × Hieff Canace® Plus PCR Master Mix (YEASEN). The PCR products were examined by 1.5% agarose gel electrophoresis and then sent for Sanger sequencing. Primer sequences were listed in Table S2B.

**Generation of CHO^td^DBA^GFP^ and A9^td^H1^GFP^ cells**

To induce cell-fusion, equal numbers of CHO^td^ (or A9^td^) and DBA-puro-GFP mESCs (or H1-puro-GFP hESCs) were mixed in presence of polyethylene glycol (PEG) (Roche) as directed by the manufacturer’s instructions, according to a previous report(Cowan et al., 2005). The cell fusion mixtures were further cultured under standard conditions for maintenance as described above. 5 – 7 days after PEG treatment, cells exhibiting dual-positive fluorescence for both GFP and tdTomato were isolated through cell sorting. The sorted single cells were cultured into independent hybrid cell lines.

**Micro-cell Mediated Chromosome Transfer (MMCT)**

MMCT was performed as described(Liskovykh et al., 2016, Sanford and Stubblefield, 1987). In brief, CHO^td^DBA^GFP^ or A9^td^H1^GFP^ cells were treated with Demecolcine solution (Sigma-Aldrich) (75 ng/mL) for 72 h to induce micronucleation. Then, cells were harvested via trypsinization, washed, and treated with Latrunculin B (0.2 μM for CHO^td^DBA^GFP^; 10μM for A9^td^H1^GFP^) to disrupt cytoskeletal integrity. Microcells were isolated by Percoll gradient centrifugation (19,000 rpm, 34°C, 80 min), followed by sequential filtration through polycarbonate membranes (8, 5, and 3 μm). Purified microcells were resuspended in ice-cold cell fusion buffer (CFB).

C57BL/6 mESCs were trypsinized, adjusted to a concentration of 2 x 10^5^ cells/25 µl, and mixed with microcells in CFB. HVJ-E suspension (10 µL) was added to the mixture, incubated on ice for 5 min, and fusion was triggered at 37°C for 15 min. Post-fusion, cells were cultured in 2iL medium. Puromycin selection (1 µg/mL) was performed 48 h after microcell fusion, and puro-resistant colonies were picked after 7 to 10 days.

**Tetraploid compensation**

For preparation of tetraploid embryos, super ovulated B6D2F1 females (8 weeks old) were mated with B6D2F1 males (2 – 6 months old). Zygotes were collected 22-24 hours after hCG injection. After 24 hr culture, E1.5 embryos at the two-cell stage were placed in the microdroplets containing 0.3 M Mannitol with 0.3% bovine serum albumin (BSA, Sigma-Aldrich) and fused with Electro Cell Fusion CFB16-HB instrument (BEX Ltd., Japan) with 0.1 cm Micro fusion slide (BTX 450-1). At first alternating current with 15 V was applied for 15 s to turn the two-cell stage embryos into the right position for fusion. After embryos had reached this position (cleaving line parallel to the electrodes), fusion pulses were started (direct current, 100 V). Time between the fusion pulses was 40 μs. The number of pulses was 2. After pulsation the embryos were washed twice immediately in KSOM+AA with D-Glucose (Millipore) and were incubated at 37°C under 5% CO_2_ for 1h. The fused embryos were washed 6-8 times in KSOM+AA with D-Glucose (Millipore) and were incubated at 37°C under 5% CO_2_.

For tetraploid compensation, ES cells were aggregated and cultured with denuded 4-cell stage mouse tetraploid embryos. In detail, clumps of loosely connected ES cells (15 – 20 cells in each) from short trypsin-treated Day2 ES cultures were transferred into microdroplets of KSOM+AA with D-Glucose under mineral oil. Each clump was placed in a depression in the microdroplet. Meanwhile, 30-50 embryos were briefly incubated in Tyrode's solution until dissolution of their zona pellucida. Two embryos were placed on one ES clump. All aggregates were assembled in this manner and cultured overnight at 37°C under 5% CO_2_.

For mouse embryo transfer, ICR female mice (8 weeks old) in the stage of estrus were selected as recipients and mated with vasectomized ICR males (3 – 12 months old) overnight to induce pseudopregnancy. Approximately 15 – 20 blastocysts were transferred into the uterine horn of a 2.5 dpc pseudo pregnant recipient.

**Immunofluorescence**

Cells were fixed with 4% paraformaldehyde (PFA) for 10 min at room temperature, washed three times with PBS, permeabilized and blocked with 0.1% Triton X-100 with 10% normal donkey serum (Jackson ImmunoResearch Laboratories) in PBS for 1 h. Staining with primary antibodies were performed overnight at 4 °C in 10% normal donkey serum and 0.1% Triton X-100. After three washes in PBS, secondary antibodies and DAPI were applied for 1 h at room temperature. Coverslips were then mounted on glass slides using SlowFade Diamond Antifade Mountant (Invitrogen). The following primary antibodies were used: anti-mouse OCT3/4 (mouse monoclonal, 1:100; sc-5729, Santa Cruz), anti-mouse SOX2 (Rabbit polyclonal, 1:100; AB5603, Millipore), anti-mouse SSEA1 (mouse monoclonal, 1:100; MAB4301, Millipore), Anti-phosphorylated histone H2AX (Ser139) (mouse monoclonal, 1:1000; 05-636, Millipore).

Mouse tissues were fixed with 4% PFA for 24 h at 4°C, dehydrated in 15% and 30% sucrose overnight at 4°C respectively until the tissues sank to the bottom of the tube, then embedded in O.C.T. compound at -30°C and stored at -80°C until further processing. Frozen tissues were cut on a cryostat (Leica CM1950) into 8 μm-thick sections for immunostaining. The slides were washed three times with PBS. After permeabilization and blocking with 0.1% Triton X-100 with 10% normal donkey serum in PBS for 1 h at room temperature. Slides were incubated with primary antibodies overnight at 4°C, secondary antibodies for 1 h at room temperature, and finally DAPI. The images of stained slides were taken by Nikon A1 confocal microscope. All quantitative analysis of immunostained sections were carried out using ImageJ. The following primary antibodies were used: Anti-mouse CD45 (Rat monoclonal, 1:100; 550539, BD Bioscience), Anti-mouse CD68 (Rat monoclonal, 1:100; MCA1957T, BIO-RAD).

**Metaphase spread preparation**

Mouse ES cells were treated for 1.5 – 2 h with 200 ng/ml Demecolcine solution (Sigma-Aldrich), harvested by trypsinization and centrifugation. Cell pellets were resuspended in 200 μl PBS followed by adding 2 ml of hypotonic 75 mM KCl/PBS (7:3) solution dropwise while gently vortexing, incubated for 10 min at 37 °C water bath and fixed using freshly prepared, ice-cold methanol/acetic acid (3:1). Cells were subsequently dropped onto slides and air dried for further processing.

**DNA fluorescence in situ hybridization (FISH)**

FISH probes (MetaSystems) were applied to metaphase spreads dropped onto slides. Slides were sealed with a coverslip and denatured at 75°C for 2 min and incubated in a humidified chamber at 37°C overnight for hybridization. Slides were washed with 0.4× SSC at 72°C for 2 min and 2× SSC, 0.05% Tween-20 at room temperature for 30 s. Then rinsed briefly in distilled water to avoid crystal formation. Slides were air-dried, stained with DAPI and mounted with SlowFade Diamond Antifade Mountant (Invitrogen).

For immunofluorescence combined with DNA FISH (IF-FISH), the immunofluorescence procedure was performed first. In detail, cell suspensions were concentrated to 1 × 10^6^ cells per ml in PBS and centrifuged onto glass slides using a Cytospin centrifuge (CenLee), fixed with 4% formaldehyde for 10 min, washed three times with PBS, permeabilized in 0.3% Triton X-100 in PBS for 5 min, incubated in Triton Block (0.2 M glycine, 2.5% fetal bovine serum, 0.1% Triton X-100, PBS) for 1 h at room temperature. Staining with primary antibodies was performed overnight at 4 °C. The following primary antibodies were used at the indicated dilutions in Triton Block: Anti-phosphorylated histone H2AX (Ser139) (Rabbit polyclonal, 1:400; 2577, Cell Signaling Technology), Anti-CENP-A (C51A7) (Rabbit monoclonal, 1:200; 2048, Cell Signaling Technology). Cells were washed with 0.1% Triton X-100 in PBS, incubated with 1:1,000 dilutions of Alexa Fluor-conjugated donkey anti-rabbit or donkey anti-mouse secondary antibodies (Jackson ImmunoResearch Laboratories) for 1 h at room temperature, and washed with 0.1% Triton X-100 in PBS. Immunostained cells were fixed with freshly prepared, ice-cold methanol/acetic acid (3:1) for 15 min at room temperature. Slides were rinsed with 80% ethanol and air-dried before proceeding to the FISH protocol. The following FISH probes were used: XCP Y green (D-0324-050-FI, Metasystem), XMP Y green (D-1421-050-FI, Metasystem), XMP X orange (D-1420-050-OR, Metasystem).

**Real-time genomic DNA PCR**

Real-time genomic DNA PCR was performed as described(D'Hulst et al., 2013). In brief, genomic DNA was digested with EcoRI (New England Biolabs). The *Omp* gene on mouse chr7 was selected as reference gene due to no aneuploidy was detected for this chromosome in C57 XY^H^ mESCs (Figure 3E). To assess the retention rate of the human chrY, we designed specific primers targeting exonic regions of UTY gene. The primers used for qPCR are provided in Table S2C. The qPCR mixture (20 μl) contained 10 μl Taq Pro Universal SYBR qPCR Master Mix (Vazyme), 0.4 μl of each primer (at 10 pM), 6.5 μl of ddH2O, and 3 μl of digested genomic ESC DNA. Reaction was run on a Bio-rad CFX Opus 96 using the following cycling conditions: an initial denaturation step at 95 °C for 5 min, followed by a 39 cycles amplification step of 95 °C for 15 s, 60 °C for 15 s, and 72 °C for 15s and a final cooling step at 40 °C for 30 s.

**Droplet digital PCR (ddPCR)**

ddPCR was performed as described(Codner et al., 2016). In brief, the *Sod2* gene on mouse chr17 and the *RPPH1* gene on human chr14 were selected as reference gene. To assess the copy number of chrY, we designed specific primers targeting puromycin. The primers and probes used for ddPCR are provided in Table S2D. Genomic DNA was digested with HindIII (New England Biolabs). The ddPCR mixture (20 μl) contained 60ng genomic DNA, 1x ddPCR Supermix for probes (Bio-Rad), 225 nM of each primer and 50 nM of each probe (one VIC-labelled probe for the reference gene assay and one 6-FAM-labelled for Y chromosome target assay). Reaction was amplified on the Bio-Rad C1000 Touch thermocycler (95 °C for 10 min, followed by 40 cycles of 94 °C for 30 s and 58 °C for 60 s, with a final step of 98 °C for 10 min). The plate containing the droplet amplicons was subsequently loaded into the QX200 Droplet Reader (Bio-Rad). The Y chromosome target was amplified in parallel with a reference gene assay, a copy number variation experiment with the reference set at 2 copies (euploid, CNV2) on the Bio-Rad QX200 ddPCR system (Bio-Rad, CA) was performed.

**Whole-genome sequencing (WGS) analysis**

Genomic DNA was isolated from ~1×10^6^ cells or 10 – 50 mg tissues using the DNeasy Blood and Tissue Kit (QIAGEN). WGS was performed by MGI2000. The raw data were qualified by SOAPnupk (v2.1.6)(Chen et al., 2017) with default parameters. Qualified reads were mapped to the mouse genome (mm39) or combined reference genome (mm39 and the Y chromosome of T2T-CHM13 v2.0) by BWA mem (v0.7.12)(Li and Durbin, 2009). Aligned sequencing reads were processed using Samtools (v1.12)(Danecek et al., 2021), and duplicate reads were flagged using Sambamba (v0.8.1)(Tarasov et al., 2015). Sequencing depth was calculated at 100 base pair windows using Mosdepth (v0.3.1)(Pedersen and Quinlan, 2018). Control-FREEC (v11.6)(Boeva et al., 2012) was used to perform copy-number variation analysis using default parameters.

To identify the genome wide variants with high confidence, we conducted single nucleotide variation and InDels calling using Mutect2 (v4.2.0).

**RNA isolation and RNA-seq**

Total RNA of each sample was extracted using Trizol (Invitrogen) according to the standard protocol. High-throughput mRNA sequencing was carried out using Illumina Novaseq 6000 and ~22 million reads were produced for each library. FastQC (v0.11.8) and Trimmomatic (v0.39)(Bolger et al., 2014) were used for quality control. Qualified reads were mapped to the mouse genome (mm39) or combined reference genome (mm39 and the Y chromosome of T2T-CHM13 v2.0) by STAR (v2.7.10a)(Dobin et al., 2013) with two pass models. Differentially expressed genes were analyzed using DESeq2.

**Differentially expressed genes (DEGs) analysis**

A comparative analysis was conducted on the gene expression patterns in 8 tissues procured from six C57 XY^H^ GFP^+^ mice and five male C57BL/6 mice aged P1 to P15. Initially, marker genes were identified within tissues of male C57BL/6 mice by selecting those with a log2(Fold-change) exceeding 0.25 and adjusted p-value less than 0.05. Subsequently, the expression patterns of these identified genes were examined in tissues from both male C57BL/6 mice and C57 XY^H^ GFP^+^ mice. The DEGs of each tissue was visualized in Table S3.

**Gene function annotation of differentially expressed genes**

We calculated differentially expressed genes (DEGs) for each cell type between the C57 XY^H^ GFP^+^ mice and male C57BL/6 mice. Genes with adjusted p-values less than 0.05 and absolute log2(Fold-Change) greater than 0.25 were selected as significantly different genes within each tissue. Then, functional annotation of differentially expressed genes in each tissue was performed using the enrichGO function from the clusterProfiler (v4.14.0)(Wu et al., 2021). Clustered GO terms with significantly low p-values (<0.05) and a gene count greater than or equal to 5 were considered as functionally enriched entries. The GO analysis result of each tissue was visualized in Table S4.

**Statistics**

All values are presented as mean ± SEM. from at least three independent experiments. Unpaired Student’s *t*-test (two tailed), Repeated Measures ANOVA and log-rank test were used to evaluate statistical significance (**P* < 0.05, ***P* < 0.01, ****P* < 0.001, *****P* < 0.0001). Randomization was used in all experiments. Data visualization and analysis were performed using GraphPad Prism version 9.0 (GraphPad Software) and Microsoft Excel (Microsoft 365).

**References**

BOEVA, V., POPOVA, T., BLEAKLEY, K., CHICHE, P., CAPPO, J., SCHLEIERMACHER, G., JANOUEIX-LEROSEY, I., DELATTRE, O. & BARILLOT, E. 2012. Control-FREEC: a tool for assessing copy number and allelic content using next-generation sequencing data. *Bioinformatics,* 28**,** 423-425.

BOLGER, A. M., LOHSE, M. & USADEL, B. 2014. Trimmomatic: a flexible trimmer for Illumina sequence data. *Bioinformatics,* 30**,** 2114-2120.

CHEN, Y., CHEN, Y., SHI, C., HUANG, Z., ZHANG, Y., LI, S., LI, Y., YE, J., YU, C., LI, Z., ZHANG, X., WANG, J., YANG, H., FANG, L. & CHEN, Q. 2017. SOAPnuke: a MapReduce acceleration-supported software for integrated quality control and preprocessing of high-throughput sequencing data. *Gigascience,* 7.

CHI, L., FAN, B., FENG, D., CHEN, Z., LIU, Z., HUI, Y., XU, X., MA, L., FANG, Y., ZHANG, Q., JIN, G., LIU, L., GUAN, F. & ZHANG, X. 2017. The Dorsoventral Patterning of Human Forebrain Follows an Activation/Transformation Model. *Cerebral Cortex,* 27**,** 2941-2954.

CODNER, G. F., LINDNER, L., CAULDER, A., WATTENHOFER-DONZE, M., RADAGE, A., MERTZ, A., EISENMANN, B., MIANNE, J., EVANS, E. P., BEECHEY, C. V., FRAY, M. D., BIRLING, M.-C., HERAULT, Y., PAVLOVIC, G. & TEBOUL, L. 2016. Aneuploidy screening of embryonic stem cell clones by metaphase karyotyping and droplet digital polymerase chain reaction. *Bmc Cell Biology,* 17.

COWAN, C. A., ATIENZA, J., MELTON, D. A. & EGGAN, K. 2005. Nuclear reprogramming of somatic cells after fusion with human embryonic stem cells. *Science,* 309**,** 1369-1373.

D'HULST, C., PARVANOVA, I., TOMOIAGA, D., SAPAR, M. L. & FEINSTEIN, P. 2013. Fast Quantitative Real-Time PCR-Based Screening for Common Chromosomal Aneuploidies in Mouse Embryonic Stem Cells. *Stem Cell Reports,* 1**,** 350-359.

DANECEK, P., BONFIELD, J. K., LIDDLE, J., MARSHALL, J., OHAN, V., POLLARD, M. O., WHITWHAM, A., KEANE, T., MCCARTHY, S. A., DAVIES, R. M. & LI, H. 2021. Twelve years of SAMtools and BCFtools. *Gigascience,* 10.

DOBIN, A., DAVIS, C. A., SCHLESINGER, F., DRENKOW, J., ZALESKI, C., JHA, S., BATUT, P., CHAISSON, M. & GINGERAS, T. R. 2013. STAR: ultrafast universal RNA-seq aligner. *Bioinformatics,* 29**,** 15-21.

HUANG, J., HE, B., YANG, X., LONG, X., WEI, Y., LI, L., TANG, M., GAO, Y., FANG, Y., YING, W., WANG, Z., LI, C., ZHOU, Y., LI, S., SHI, L., CHOI, S., ZHOU, H., GUO, F., YANG, H. & WU, J. 2024. Generation of rat forebrain tissues in mice. *Cell,* 187.

LI, H. & DURBIN, R. 2009. Fast and accurate short read alignment with Burrows-Wheeler transform. *Bioinformatics,* 25**,** 1754-1760.

LISKOVYKH, M., LEE, N. C. O., LARIONOV, V. & KOUPRINA, N. 2016. Moving toward a higher efficiency of microcell-mediated chromosome transfer. *Molecular Therapy-Methods & Clinical Development,* 3.

MICUTKOVA, L., HERMANN, M., OFFTERDINGER, M., HESS, M. W., MATSCHESKI, A., PIRCHER, H., MUECK, C., EBNER, H.-L., LAICH, A., FERRANDO-MAY, E., ZWERSCHKE, W., HUBER, L. A. & JANSEN-DUERR, P. 2012. Analysis of the cellular uptake and nuclear delivery of insulin-like growth factor binding protein-3 in human osteosarcoma cells. *International Journal of Cancer,* 130**,** 1544-1557.

PEDERSEN, B. S. & QUINLAN, A. R. 2018. Mosdepth: quick coverage calculation for genomes and exomes. *Bioinformatics,* 34**,** 867-868.

SANFORD, J. A. & STUBBLEFIELD, E. 1987. General protocol for microcell-mediated chromosome transfer. *Somatic cell and molecular genetics,* 13**,** 279-84.

TARASOV, A., VILELLA, A. J., CUPPEN, E., NIJMAN, I. J. & PRINS, P. 2015. Sambamba: fast processing of NGS alignment formats. *Bioinformatics,* 31**,** 2032-2034.

WU, T., HU, E., XU, S., CHEN, M., GUO, P., DAI, Z., FENG, T., ZHOU, L., TANG, W., ZHAN, L., FU, X., LIU, S., BO, X. & YU, G. 2021. clusterProfiler 4.0: A universal enrichment tool for interpreting omics data. *Innovation,* 2.

**Supplemental figures**

**
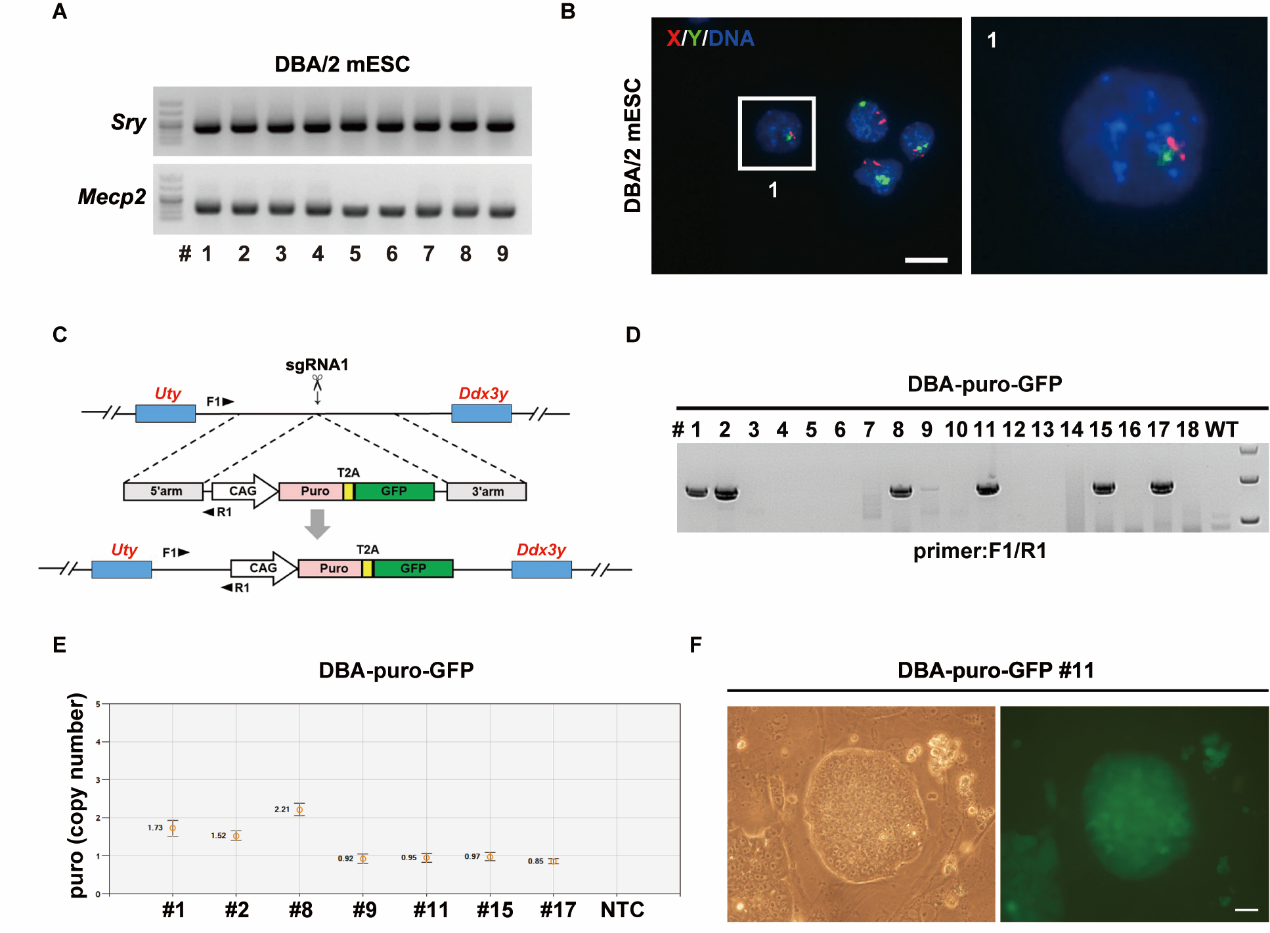
**

**Figure S1 Generation of intraspecies Y chromosome donor cell line**

1. Genomic DNA PCR results of nine DBA/2 ESC clones. *Sry* and *Mecp2* are located on chromosome Y and X, respectively.
2. Representative DNA-FISH analysis of DBA/2 ESC clone. Green, whole-chromosome probe for mouse Y chromosome; red, whole-chromosome probe for mouse X chromosome; blue, Hoechst-labeled DNA. Scale bar, 10 μm.
3. Schematic representation of the genome editing strategy for CAG-puro-GFP knock-in in mouse chrY. The positions of the designed primers for genomic PCR are shown as triangles.
4. Genomic DNA PCR results of 18 colonies retrieved from CAG-puro-GFP knock-in. The primers used are indicated in (C).
5. Copy number of puromycin in seven DBA-puro-GFP ESC clones indicated in (D). NTC, No Template Control.
6. Morphology of DBA-puro-GFP ESC line #11. DBA-puro-GFP #11 expressed GFP. Scale bar, 100 μm.


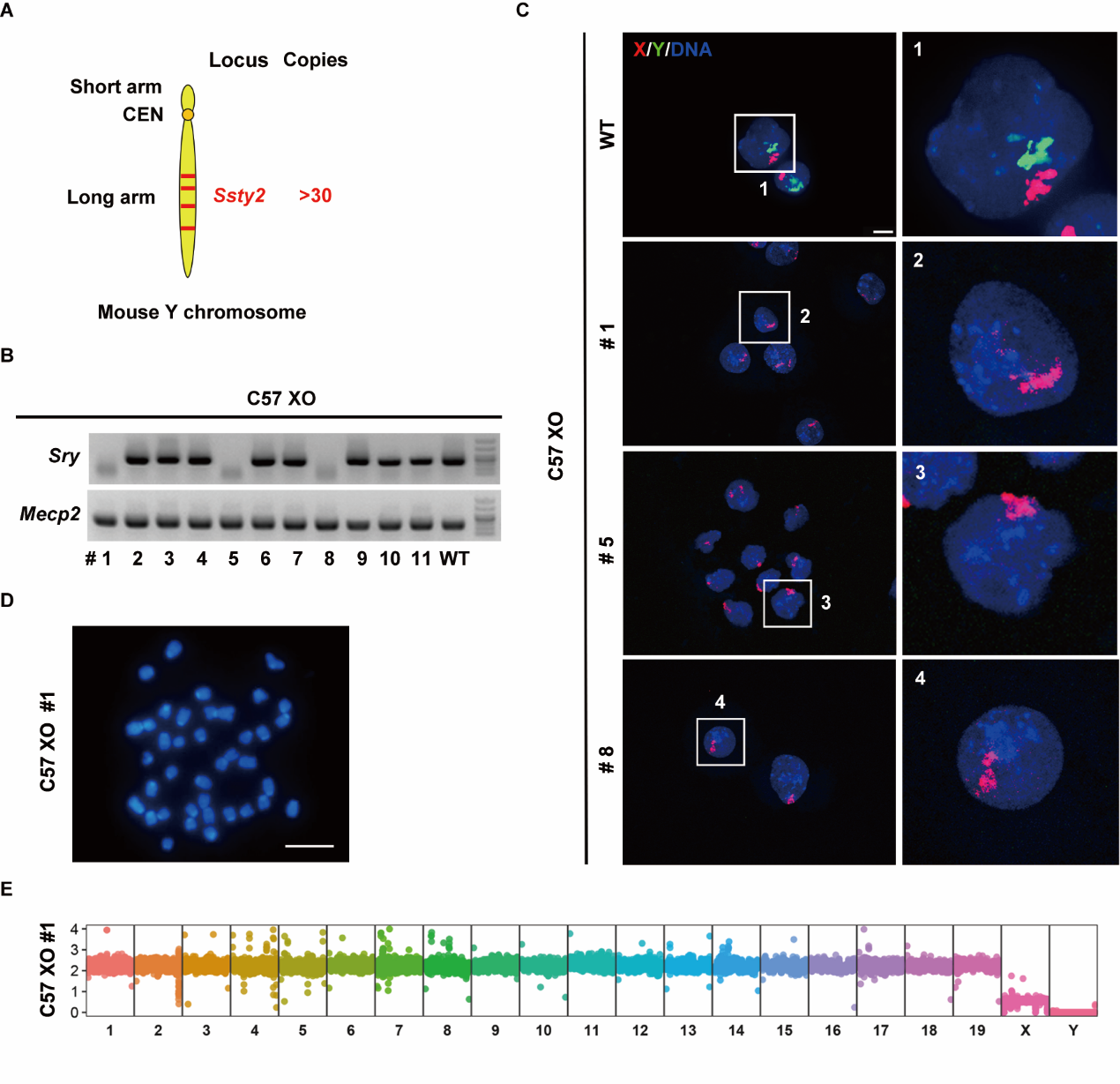


**Figure S2 Generation of Y chromosome recipient cell line**

1. Targeted gene loci in mouse Y chromosome: *Ssty2*, scattered in the long arm. CEN, centromere.
2. Genomic DNA PCR results of 11 colonies retrieved from C57BL/6 chrY elimination. *Sry* and *Mecp2* are located on chromosome Y and X, respectively.
3. Representative DNA-FISH analysis of male C57 ESC clone and C57 XO ESC clones indicated in (B). Green, whole-chromosome probe for mouse Y chromosome; red, whole-chromosome probe for mouse X chromosome; blue, Hoechst-labeled DNA. Scale bar, 10 μm.
4. Karyotyping of C57 XO ESC line #1 showed 39 instead of 40 chromosomes. Scale bar, 10 μm.
5. WGS showed chrY elimination of C57 XO ESC line #1. The C57 XO #1 showed one copy of the chrX with the chrY absent. Vertical axis, copy number; horizontal axis, chromosome number.


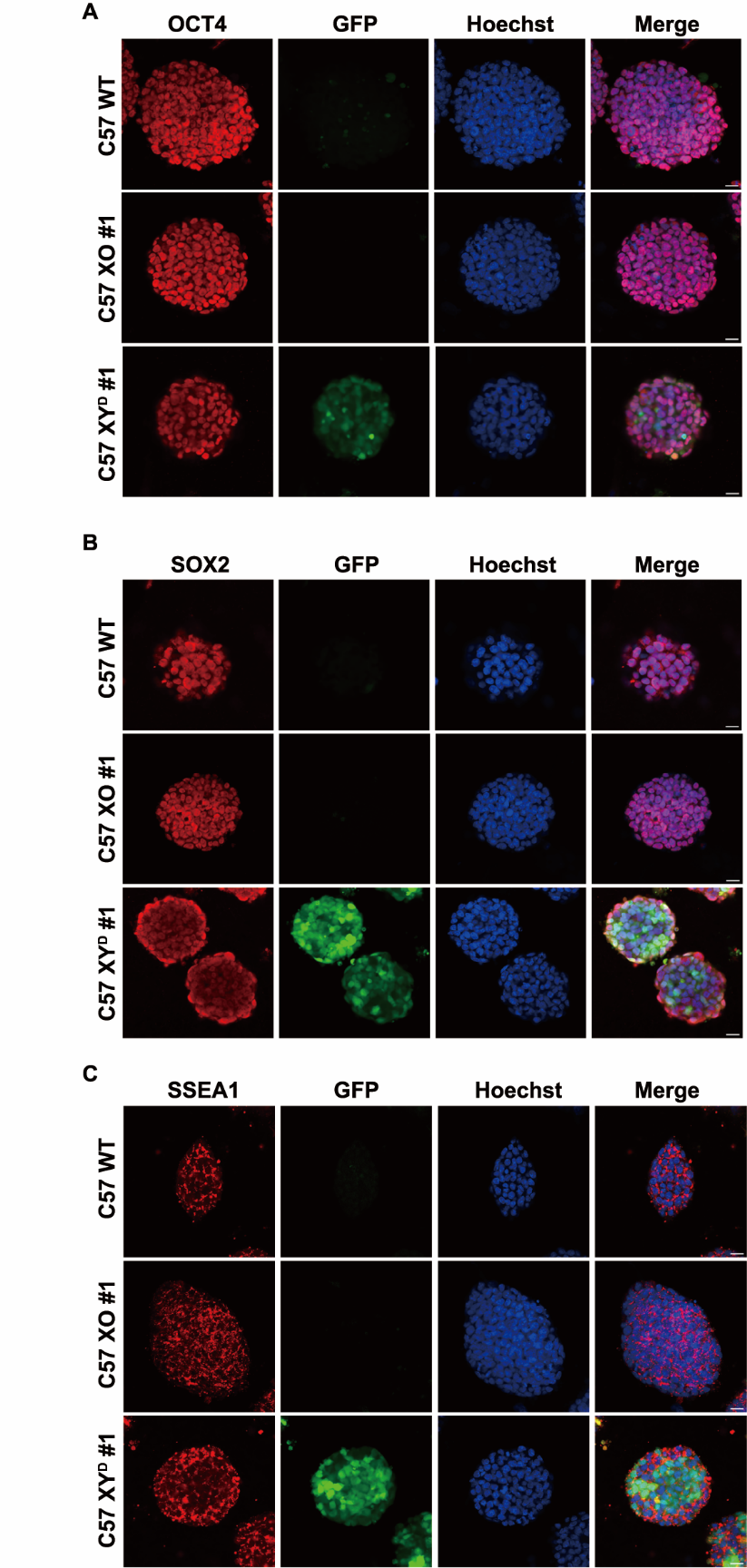


**Figure S3 Immunofluorescence staining analysis of pluripotency markers in C57 XY^D^ mESCs**

A-C. Immunostaining of pluripotency marker OCT4, SOX2 and SSEA1 in wild-type C57BL/6 (C57 WT), C57 XO #1 and C57 XY^D^ #1 ESCs. Scale bars, 20 μm.


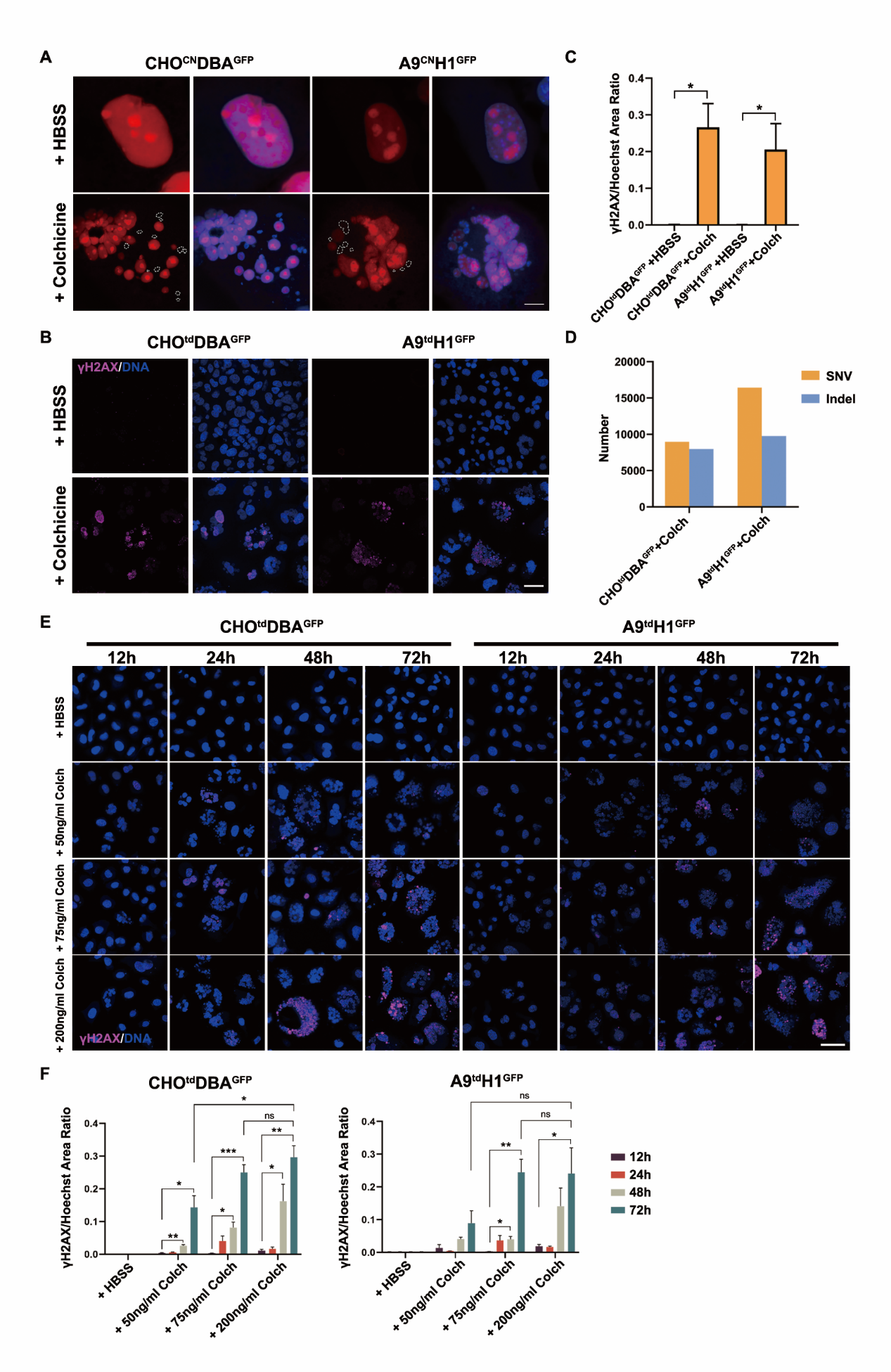


**Figure S4 Nuclear envelope instability and DNA damage during MMCT**

A – D. Cells treated with 75 ng/ml colchicine for 72 h.

1. Parts of micronuclei in HBSS-treated (+ HBSS) and colchicine-treated (+ Colchicine) CHO^CN^DBA^GFP^ and A9^CN^H1^GFP^ cells lose mCherry–NLS, which were marked with dashed lines. Scale bar, 20 μm.
2. Representative immunofluorescence images of HBSS-treated (+ HBSS) and colchicine-treated (+ Colchicine) CHO^td^DBA^GFP^ and A9^td^H1^GFP^ cells, stained with Hoechst (blue) and DNA damage marker γH2AX (pink). Scale bar, 50 μm.
3. The ratio of γH2AX-positive area to Hoechst-stained nuclear area indicated in (B) (n = 5 slices per group). Data are presented as mean ± SEM. *p < 0.05, unpaired two-tailed Student's t test.
4. Comparison of number of detected SNVs and indels. The number of SNVs for colchicine-treated CHO^td^DBA^GFP^ and colchicine-treated A9^td^H1^GFP^ were 8956 and 16422, respectively. The number of indels for colchicine-treated CHO^td^DBA^GFP^ and colchicine-treated A9^td^H1^GFP^ were 7982 and 9766, respectively.
5. Representative immunofluorescence images of CHO^td^DBA^GFP^ and A9^td^H1^GFP^ cells following colchicine treatment at varying concentrations (0, 50, 75, and 200 ng/ml) and exposure times (12, 24, 48, and 72 h), stained with Hoechst (blue) and DNA damage marker γH2AX (pink). The 0 ng/ml group is labeled as “+ HBSS”. Scale bar, 50 μm.
6. The ratio of γH2AX-positive area to Hoechst-stained nuclear area indicated in (E) (n = 3 slices per group). Data are presented as mean ± SEM. *p < 0.05, **p < 0.01, ***p < 0.001, unpaired two-tailed Student's t test. ns, no significance.


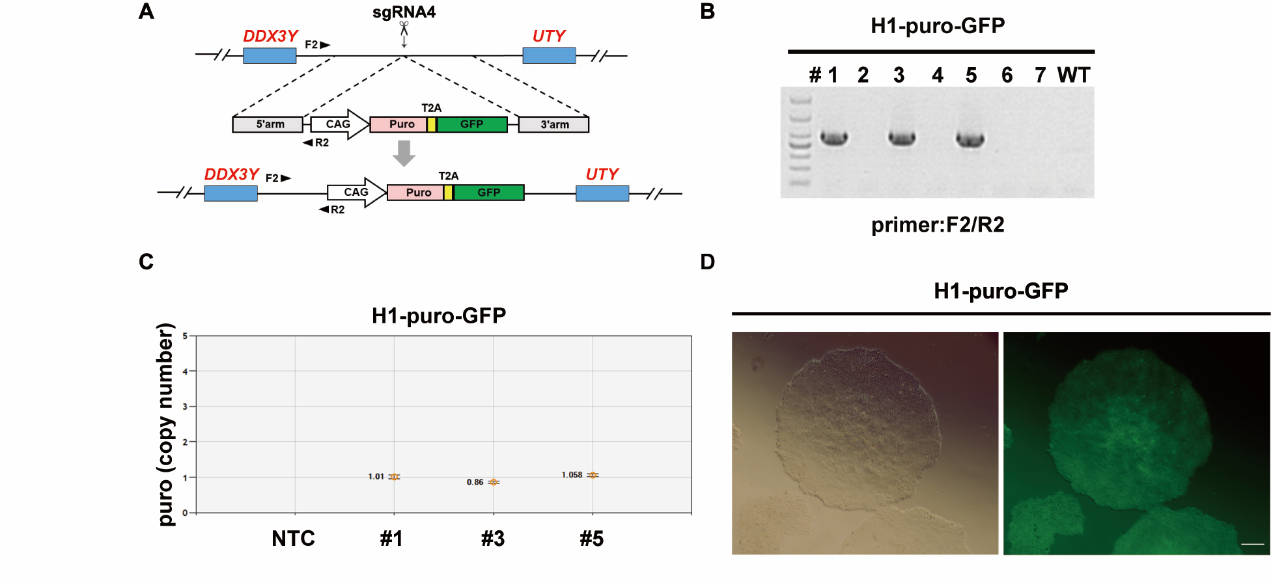


**Figure S5 Generation of interspecies Y chromosome donor cell line**

1. Schematic representation of the genome editing strategy for CAG-puro-GFP knock-in in human chrY. The positions of the designed primers for genomic PCR are shown as triangles.
2. Genomic DNA PCR results of 7 colonies retrieved from CAG-puro-GFP knock-in. The primers used are indicated in (A). WT, H1 human ESC.
3. ddPCR results of three H1-puro-GFP ESC clones. Copy number of puromycin in three H1-puro-GFP ESC clones indicated in (B). NTC, No Template Control.
4. Morphology of H1-puro-GFP ESC line. H1-puro-GFP ESCs expressed GFP. Scale bar, 250 μm.


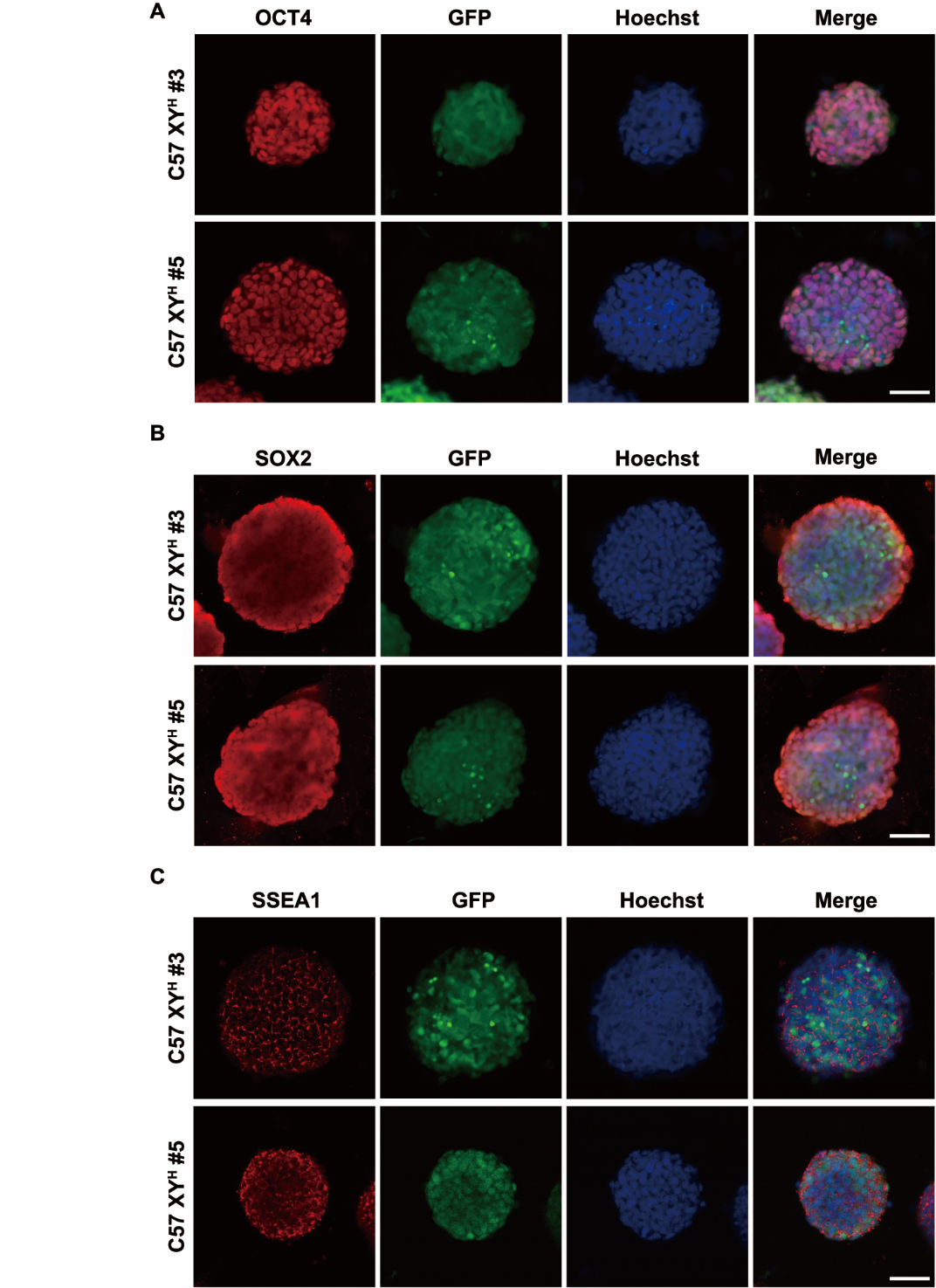


**Figure S6 Immunofluorescence staining analysis of pluripotency markers in C57 XY^H^ mESCs**

A-C. Immunostaining of pluripotency marker OCT4, SOX2 and SSEA1 in C57 XY^H^ #3 and C57 XY^H^ #5 ESCs. Scale bars, 50 μm.


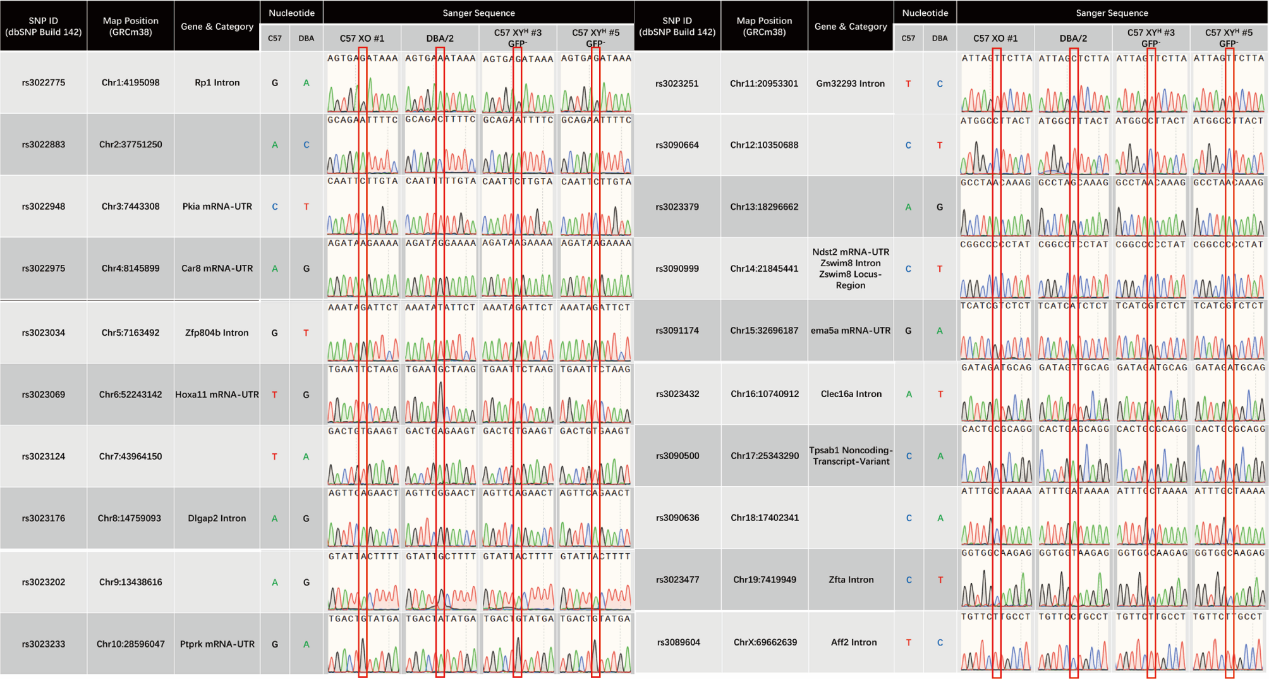


**Figure S7 Genotyping of SNP loci with sequence variations between C57BL/6 and DBA/2 mice.**

The table lists SNP loci with sequence variations between C57BL/6 and DBA/2 mice. Nucleotide differences are highlighted in red boxes. Representative genotyping results are shown for individual mice from four strains: C57 XO #1, DBA/2, C57 XY^H^ #3 GFP^-^, and C57 XY^H^ #5 GFP^-^. Notably, C57 XY^H^ #3 GFP^-^ and C57 XY^H^ #5 GFP^-^ mice exclusively retain C57-specific alleles at all SNP loci. SNP positions are annotated with chromosome position (GRCm38 assembly) and gene categories (e.g., intronic, mRNA-UTR).


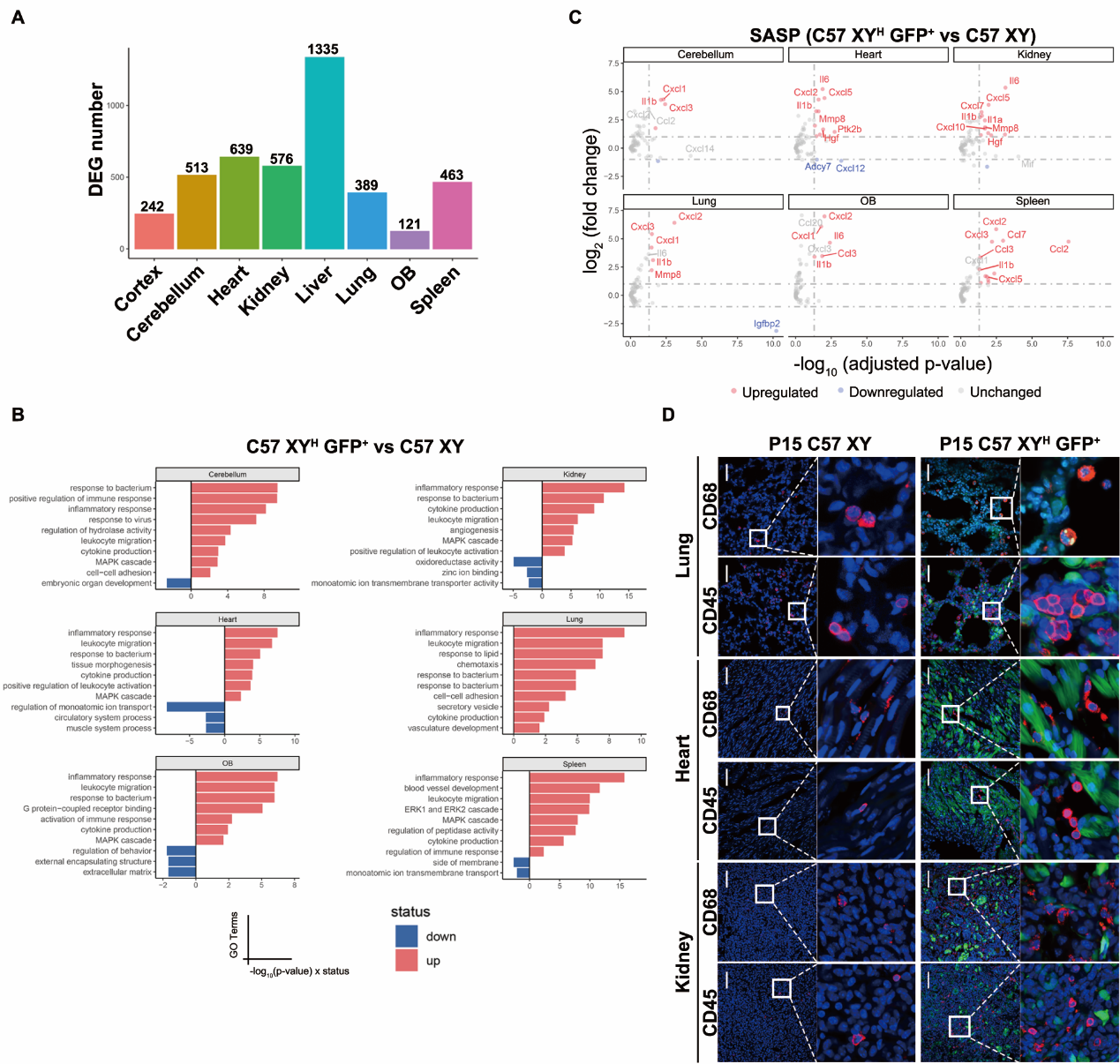


**Figure S8 Activation of inflammatory responses in C57 XYᴴ GFP^+^ mice**

1. The number of differentially expressed genes across various organs in C57 XYᴴ GFP^+^ mice compared to male C57BL/6 mice from postnatal day 1 to day 15 (n = 11). OB, olfactory bulb.
2. Gene Ontology (GO) enrichment analysis of DEGs across various tissues in C57 XYᴴ GFP^+^ mice compared to male C57BL/6 mice. OB, olfactory bulb.
3. Scatter plots showing the differential expression levels of SASP genes across various tissues in C57 XYᴴ GFP^+^ mice compared to male C57BL/6 mice. OB, olfactory bulb.
4. Various tissues of P15 male C57BL/6 and P15 C57 XYᴴ GFP^+^ mice were immunostained for CD45 or CD68 (red). Hoechst (blue) was used to label DNA. Scale bars, 50 μm.
